# Rising global population coexposure to heatwaves and landscape fire-sourced air pollution

**DOI:** 10.1126/sciadv.aea9749

**Published:** 2026-10-14

**Authors:** Yao Wu, Rongbin Xu, Bo Wen, Lidia Morawska, Michael J Abramson, Fay H. Johnston, Wenhua Yu, Gongbo Chen, Yiwen Zhang, Zhihu Xu, Wenzhong Huang, Zhengyu Yang, Pei Yu, Shuang Zhou, Yanming Liu, Tingting Ye, Xu Yue, Shanshan Li, Yuming Guo

**Affiliations:** ^1^ MEEKL-AERM, College of Environmental Sciences and Engineering, Institute of Tibetan Plateau, and Center for Environment and Health, Peking University, Beijing 100871, China.; ^2^ TUWAS-AEEEH, Tibet University, Lhasa, Xizang 850000, China.; ^3^ Climate, Air Quality Research (CARE) Unit, School of Public Health and Preventive Medicine, Monash University, Melbourne, VIC 3004, Australia.; ^4^ Chongqing Emergency Medical Center, Chongqing University Central Hospital, School of Medicine, Chongqing University, Chongqing 400044, China.; ^5^ School of Public Health and Preventive Medicine, Faculty of Medicine, Nursing and Health Sciences, Monash University, Melbourne, VIC 3004, Australia.; ^6^ School of Earth and Atmospheric Sciences, Queensland University of Technology, Brisbane, QLD 4000, Australia.; ^7^ Menzies Institute for Medical Research, University of Tasmania, Melbourne, VIC 3000, Australia.; ^8^ Department of Urban Planning and Design, the Social Infrastructure for Equity and Wellbeing (SIEW) Lab, and the Hong Kong Base of the State Key Laboratory of Subtropical Building and Urban Science, The University of Hong Kong, Hong Kong SAR, China.; ^9^ School of Statistics and Data Science, Southeast University, 211189, Nanjing, China.; ^10^ Jiangsu Key Laboratory of Atmospheric Environment Monitoring and Pollution Control, Collaborative Innovation Center of Atmospheric Environment and Equipment Technology, Joint International Research Laboratory of Climate and Environment Change, School of Environmental Science and Engineering, Nanjing University of Information Science and Technology, Nanjing 210044, China.

## Abstract

Landscape fires are increasingly threatening public health by causing substantial fire-sourced air pollution (SFAP), with risks amplified during heatwaves. We investigated the global spatial, temporal, and sociodemographic distribution of the population coexposure to heatwaves and SFAP from 2000 to 2019. We found that 52.4% of populated areas and 55.9% of the global population experienced increasing coexposure. On average, 916 million people per year were exposed to at least one coexposure day, increasing by 287 million people per decade. The global annual average exposed days per person was 1.3 days, rising from 1.0 days in 2000–2009 to 1.7 days in 2010–2019. Coexposure person-days increased by 5.5 billion per decade, of which 70.5% was attributable to increasing coexposure intensity and 29.5% to population growth. Countries with a low human development index (HDI) experienced the highest coexposure days per person and the greatest increases. These findings provide high-resolution data to inform health impact assessments and target mitigation and adaptation strategies.

## INTRODUCTION

Human-induced climate change is driving a shift in the global fire regime, transitioning from a precipitation-dominated pattern in the preindustrial era to a temperature-driven system in the 21st century (*1*). Attribution studies indicate that anthropogenic warming has contributed to more than half of the observed increase in fuel aridity during 1979–2015 and has approximately doubled cumulative forest fire area burned in the western United States relative to what would have occurred in the absence of anthropogenic climate change since the mid-1980s (*2*). This transition has increased the frequency and intensity of concurrent heatwaves and landscape fire events, resulting in more profound impacts (*3*–*7*). A record-breaking fire in 2021 burned 3.2 million ha in western Canada and United States, with heat dome accounting for 21 to 34% of the total area burned (*8*). In 2022, heatwave-fueled fires raged across Western Europe, burning up to 130,000 ha in Portugal, Spain, and France (*9*). In the Brazilian Pantanal, the 2020 fire season, exacerbated by heatwaves and droughts, resulted in total economic losses of around 3.6 billion dollars (*10*).

Heatwaves and landscape fires are intrinsically linked. Elevated temperatures, increased vapor pressure deficit, and soil moisture deficits promote fire weather (characterized by hot, dry, and windy conditions) by reducing fuel moisture and facilitating ignition and rapid spread (*11*–*13*). Conversely, landscape fires exacerbate heatwaves by releasing large amounts of heat and pollutants into the atmosphere, leading to drier conditions and higher temperatures (*14*). Also, strong winds can spread landscape fires and contribute to the development and persistence of heatwaves by supplying the flame front with fresh oxygen and tilting flames forward (*15*). These processes create a positive feedback loop in which heatwaves and fires amplify one another.

High temperatures, identified as a leading risk factor in the Global Burden of Diseases, Injuries, and Risk Factors Study (GBD), accounted for 14.1 million disability-adjusted life-years globally in 2021 (*16*). Simultaneously, global populations have also been increasingly exposed to fine particulate matter (PM_2.5_) and ozone (O_3_) air pollution from landscape fires, contributing to 1.53 million all-cause deaths per year during 2000–2019 (*17*, *18*). Compared with exposure to heatwave or landscape fire-sourced air pollution alone, coexposure to both hazards may exert greater health impacts than the sum of their individual effects (*4*). Recent studies have identified synergistic effects of heatwave and ambient air pollution, likely driven by shared pathophysiological pathways (e.g., increased oxidative stress and inflammation) (*19*, *20*). Given the high likelihood of co-occurrence and the highly oxidative and pro-inflammatory potential of landscape fire-sourced air pollution, the health risks associated with coexposure to landscape fire-sourced air pollution and heatwave may be particularly severe (*1*, *21*–*24*).

Before evaluating health impacts, a comprehensive assessment of global population coexposure to heatwave and landscape fire-sourced air pollution is essential. However, unlike the widely studied compound drought-heatwave events, compound landscape fire-heatwave events have received less scientific attention (*25*–*27*). Existing studies on their co-occurrence are largely limited to the United States (*3*, *28*–*30*). Comparably, low-income countries bear a disproportionate burden, with landscape fire-sourced PM_2.5_ concentrations four times higher than in high-income countries (*17*). Limited financial resources, reliance on climate-sensitive sectors (e.g., agriculture), high population density, and inadequate readiness for adaptation increase the vulnerability of less developed regions to the compound landscape fire-sourced air pollution and heatwave events (*31*, *32*). The lack of accurate, high-resolution assessments of population coexposure to heatwave and landscape fire-sourced air pollution across the globe may undermine climate adaptation efforts and exacerbate environmental injustices (*4*).

This study aimed to provide a global assessment of population coexposure to heatwave and landscape fire-sourced air pollution over the past two decades (2000–2019) and explore potential spatial-temporal variations and socioeconomic disparities.

## RESULTS

### Overlap between heatwave and SFAP days

During 2000–2019, the global annual average proportion of person-days exposed to substantial fire-sourced air pollution (SFAP) within heatwave days (defined by >90th percentile; 2 days) was 3.9%, while the proportion of person-days exposed to heatwave within SFAP days was 13.6%. The geographical patterns of the overlap between heatwave and SFAP varied across regions: South America, Southern Africa, Southeast Asia, and the Siberian and Far Eastern parts of Russia had higher proportions and an increasing trend of person-days exposed to SFAP among those exposed to heatwave (Fig. 1). In contrast, North America and the eastern states of Australia exhibited higher proportions of person-days exposed to heatwave among those exposed to SFAP. Increasing trends in the proportion of SFAP person-days that coincided with heatwaves particularly evident in the northern region of South America, Australia, and Eastern Europe (Fig. 1).

**Fig. 1.**
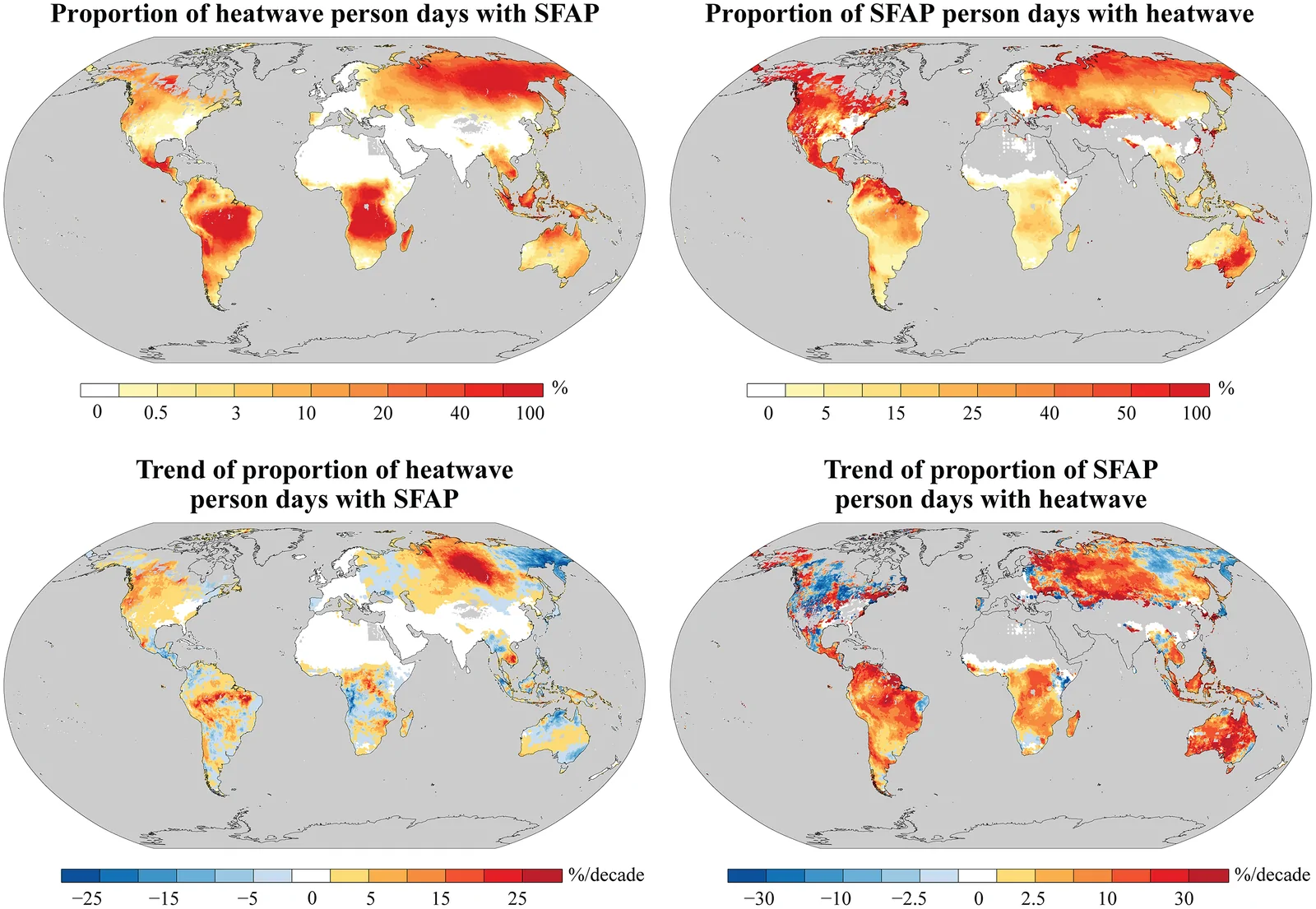
Global maps of overlap between population coexposure to heatwave and SFAP days during 2000–2019. The white areas represent grid cells where proportions were unavailable due to the absence of SFAP days. SFAP, substantial fire-sourced air pollution.

Globally, the annual average proportion of person-days exposed to heatwave among those exposed to SFAP rose from 10.0% in 2000–2009 to 17.2% in 2010–2019, reflecting an increase of 6.6 percentage points per decade (*P* < 0.001 for trend). Conversely, the proportion of person-days exposed to SFAP among those exposed to heatwave showed a modest rise, increasing from 3.4% in 2000–2009 to 4.3% in 2010–2019, equating to an increase of 0.7 percentage points per decade (*P* = 0.01 for trend). At a continental level, Africa, Asia, North America, and South America exhibited significant upward trends in the proportion of person-days exposed to heatwave among those exposed to SFAP (*P* ≤ 0.001 for trend). In contrast, the proportion of person-days exposed to SFAP among those exposed to heatwave remained relatively stable across all continents from 2000 to 2019 (Fig. 2 and table S1).

**Fig. 2.**
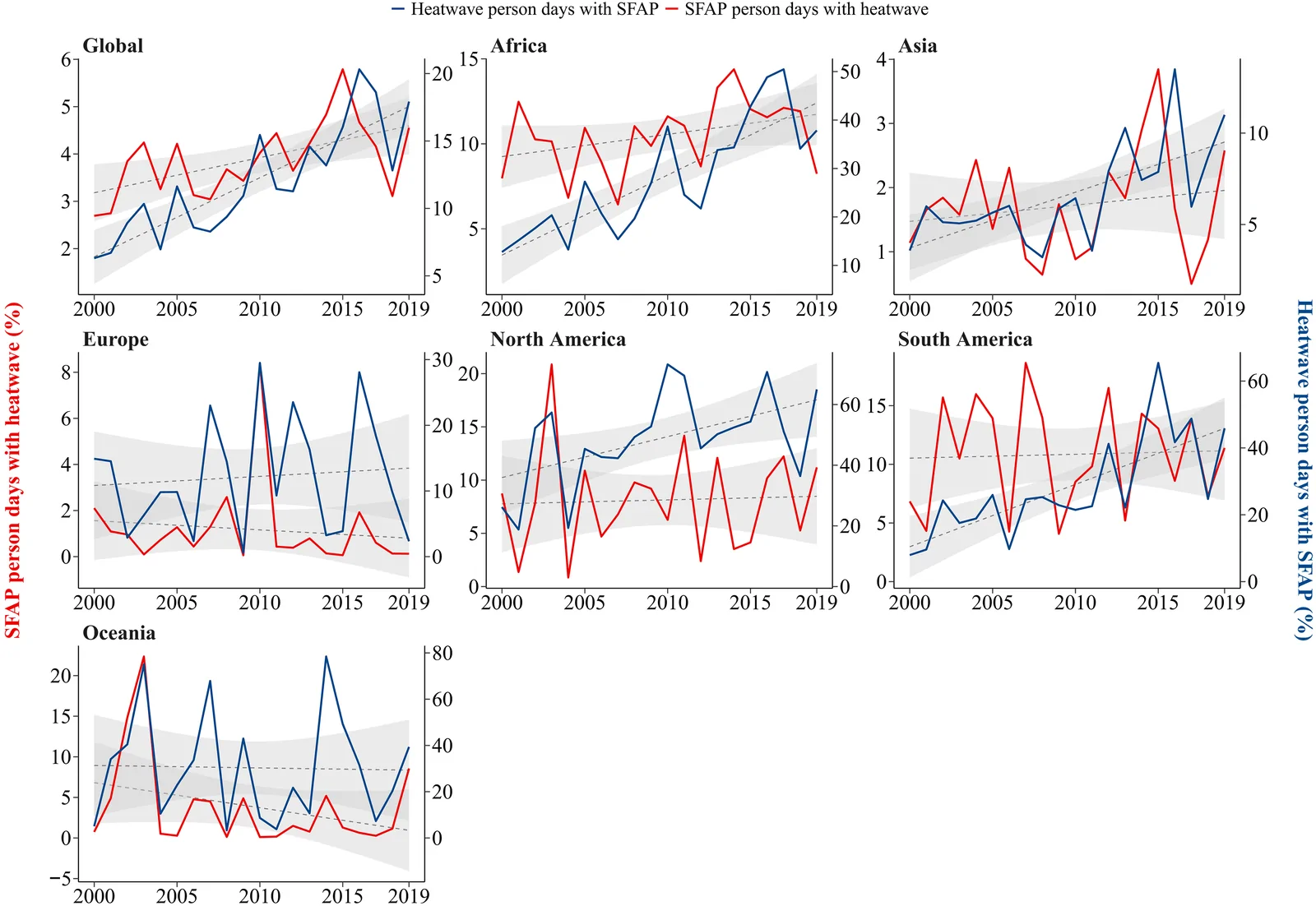
Global and continent-specific trends in proportions of person-days exposed to SFAP within heatwave-exposed days (blue lines) and heatwave within SFAP-exposed days (red lines) from 2000 to 2019. The dashed lines denote point estimates of the fitted trend by linear regression and the shaded areas denote the corresponding 95% confidence intervals.

### Temperature anomalies during SFAP days

When evaluated using local temperature anomalies, at the global level, SFAP days exhibited positive temperature anomalies compared with matched control days (fig. S1). Spatially, positive anomaly differences were observed across large parts of North America, Europe, Oceania, and southern South America, with more heterogeneous patterns in Asia. Regional summaries further confirmed that the median difference in temperature anomalies between SFAP and non-SFAP days was positive across all continents, although the magnitude varied by region.

### Temporal sequencing between heatwaves and SFAP

At the global scale, the probability of SFAP increased beginning approximately two days prior to heatwave onset, peaked on the day of heatwave onset, and returned to near-baseline levels by 2 days afterward (fig. S2). Regional heterogeneity was observed. In Africa and South America, the probability of SFAP onset increased on the day of heatwave onset and remained elevated for ∼1 day afterward, suggesting that SFAP episodes tend to coincide with or follow heatwave onset. In contrast, in Asia, Europe, North America, and Oceania, elevated SFAP probabilities were observed in the days preceding heatwave onset, indicating that SFAP episodes may develop before heatwave emergence.

### Global spatial-temporal distribution of coexposure days

Between 2000 and 2019, global trends in heatwave days and SFAP days exhibited distinct spatial patterns (fig. S3). Across grid cells, annual average number of heatwave days ranged from 17 to 37 days. In contrast, SFAP days were far more heterogeneous, with a median (interquartile range) of 0.4 (0.0 to 11.5) days per year. An increasing trend in heatwave days was observed in 87% of grid cells worldwide, particularly across South America, Africa, Southeast Asia, and Oceania. In contrast, only 28% of grid cells showed an increasing trend in SFAP days over the study period, with these increases concentrated primarily in North America.

Despite asymmetric spatial and temporal patterns in individual hazards, compound exposure was widespread. Between 2000 and 2019, 144 of the 208 countries (∼69%) experienced at least one coexposure day per year. Hotspot areas included Central America, South America, Central and Eastern Africa, Southeast Asia, and parts of Australia, Russia, and Canada (Fig. 3). Regions identified as hotspots for coexposure days also demonstrated notable decadal increases in the number of such events. At the national level, increases in the frequency of coexposure days were observed in Russia, the United States, Indonesia, and Australia during the 2010–2019 period compared to 2000–2009. Conversely, Ukraine and Moldova experienced a reduction in the frequency of these events over the same timeframe (Fig. 3).

**Fig. 3.**
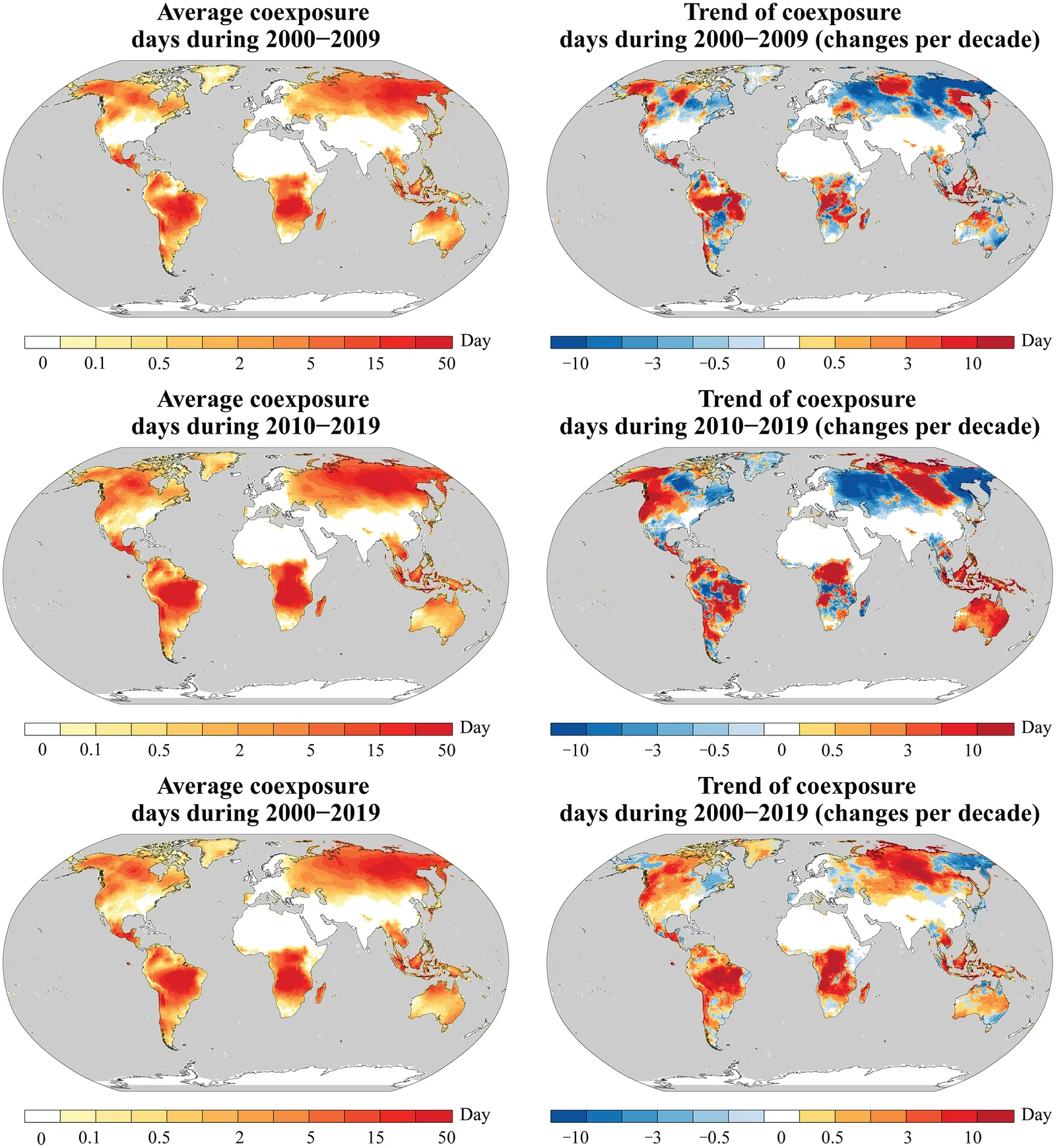
Global maps of annual average days coexposure to heatwave and SFAP in the first and second decades of 2000–2019 and the estimated trend during these periods.

### Global population coexposure to heatwave and SFAP

From 2000 to 2019, 52.4% of global populated areas and 55.9% of the global population were experiencing increasing coexposure to heatwave and SFAP. Between 2000 and 2019, an average of 916 million people per year was exposed to at least 1 day of coexposure to heatwave and SFAP globally (Table 1). The number of exposed populations increased from 761 million per year in 2000–2009 to 1072 million per year in 2010–2019, marking an increase of 287 million people per decade (*P* < 0.001 for trend). Correspondingly, the annual average number of person-days rose substantially, from 6.3 billion in 2000–2009 to 12.3 billion per year in 2010–2019, resulting in an overall average of 9.3 billion person-days per year across the study period. The annual average exposed days per person increased from 1.0 day in 2000–2009 to 1.7 days in 2010–2019, with an overall average of 1.3 days per person during the 20-year study period (Fig. 4 and Table 1).

**Table 1.** Global population coexposure to heatwave and SFAP from 2000 to 2019. HDI, human development index.

	Annual person days (×10^8^)					Annual days per person					Annual persons (×10^6^)				
	2000–2009	2010–2019	2000–2019	Change per decade	*P* for trend	2000–2009	2010–2019	2000–2019	Change per decade	*P* for trend	2000–2009	2010–2019	2000–2019	Change per decade	*P* for trend
Continent													
Africa	21.9	51.4	36.6	27.1	<0.001	2.4	4.4	3.4	1.7	<0.001	199.7	315.6	257.7	109.2	<0.001
Asia	18.0	32.0	25.0	13.4	0.016	0.5	0.7	0.6	0.3	0.041	271.0	378.8	324.9	95.4	0.017
Europe	2.0	3.5	2.8	−0.4	0.848	0.3	0.5	0.4	−0.1	0.836	53.2	54.1	53.7	−7.3	0.675
North America	11.6	18.3	15.0	6.7	0.074	2.3	3.3	2.8	1.0	0.159	118.0	156.4	137.2	38.5	0.033
South America	9.0	17.6	13.3	8.3	0.004	2.4	4.3	3.4	1.8	0.010	111.3	160.7	136.0	50.8	0.007
Oceania	0.4	0.3	0.3	0.0	0.908	1.4	0.8	1.1	−0.3	0.621	7.3	6.1	6.7	0.7	0.782
HDI group													
Low HDI	15.3	38.8	27.1	21.7	<0.001	2.5	4.7	3.6	2.0	<0.001	141.1	236.0	188.5	89.1	<0.001
Medium HDI	10.6	20.7	15.7	9.2	<0.001	0.6	1.0	0.8	0.4	0.002	117.3	161.1	139.2	45.0	0.002
High HDI	27.7	47.8	37.7	20.6	0.003	1.1	1.7	1.4	0.6	0.008	329.2	469.2	399.2	140.0	0.001
Very high HDI	9.4	15.8	12.6	3.6	0.078	0.7	1.0	0.9	0.2	0.163	172.3	204.3	188.3	13.3	0.551
Income group													
Low income	13.6	35.3	24.5	19.9	<0.001	3.0	5.9	4.4	2.6	<0.001	122.9	207.4	165.1	79.4	<0.001
Lower middle income	12.7	25.6	19.1	11.6	<0.001	0.5	0.9	0.7	0.3	0.001	164.8	239.7	202.2	72.5	0.001
Upper middle income	32.8	57.5	45.2	22.6	0.001	1.3	2.1	1.7	0.7	0.004	390.5	529.1	459.8	132.8	0.001
High income	3.9	4.8	4.3	1.0	0.456	0.3	0.4	0.4	0.1	0.635	82.3	95.2	88.7	2.6	0.895
Global	63.0	123.2	93.1	55.1	<0.001	1.0	1.7	1.3	0.6	<0.001	760.6	1071.7	916.2	287.3	<0.001

**Fig. 4.**
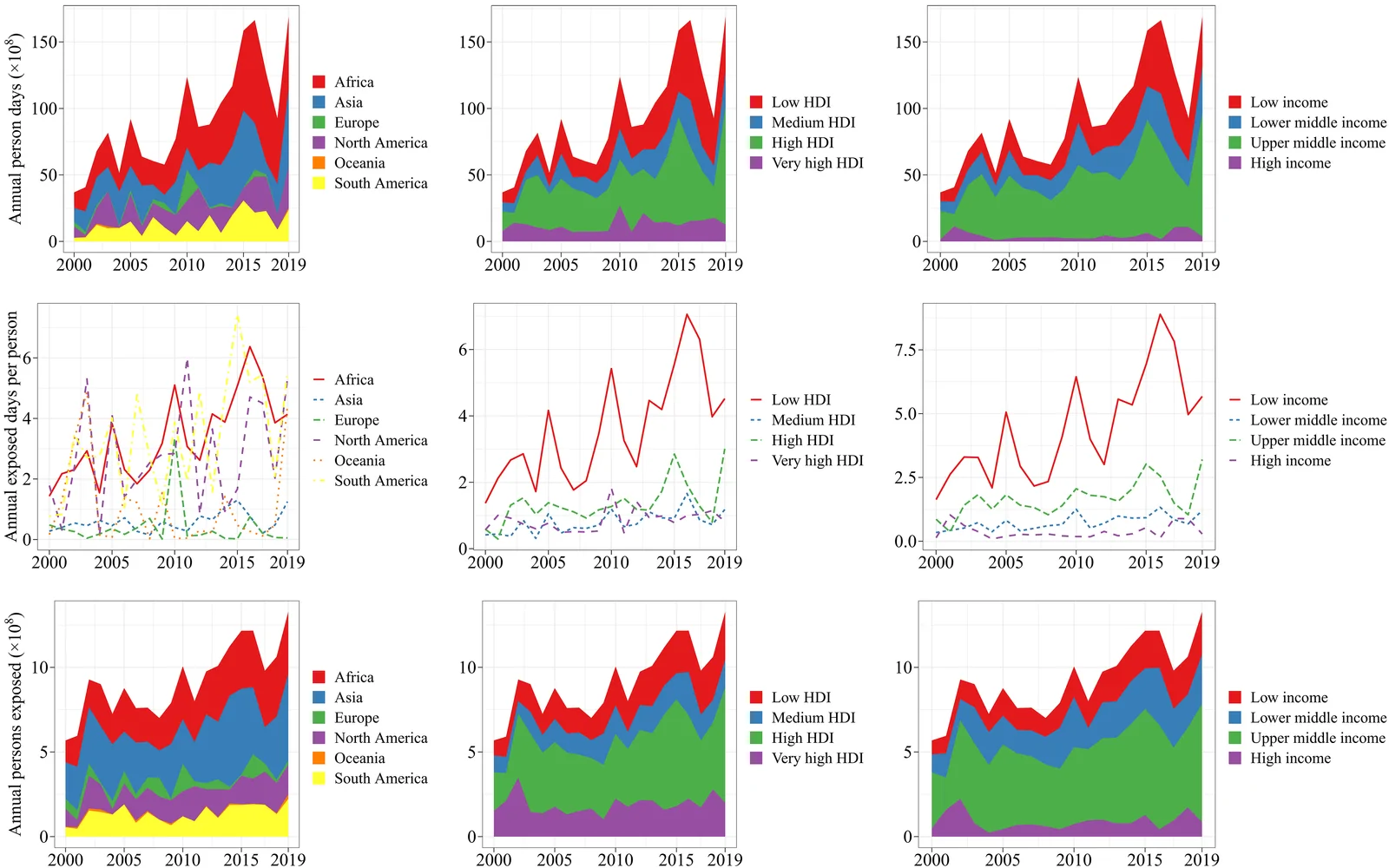
Annual population coexposure to heatwave and SFAP from 2000 to 2019 by continents, HDI, and income level. HDI, human development index.

The population exposure varied geographically. Africa experienced the largest proportion of exposed person-days (∼39% of the global total) between 2000 and 2019, followed by Asia (∼27%) (Fig. 4 and Table 1). Most continents experienced an increase in all three metrics of population exposure, with significantly increasing trends observed in Africa, Asia, and South America. Africa saw the highest increase in annual exposed persons and person-days, with an increase of 109.2 million persons and 2.7 million person-days/ decade; while South America had the highest increase in exposed days per person, with an increase of 1.8 days per person per decade. Europe and Oceania showed a slight decrease in population coexposure to heatwave and SFAP, though these changes were not statistically significant (Fig. 4 and Table 1). Trends of three metrics of population exposure to individual extreme events (heatwave or SFAP) by continent are shown in figs. S4 to S6 and tables S2 to S4.

The decomposition of the change in coexposure person-days into contributions from population growth and coexposure intensity is shown in table S5. Globally, coexposure person-days increased by 5.5 billion per decade, of which 70.5% was attributable to increasing coexposure intensity and 29.5% to population growth. Consistent with the global decomposition results, increasing exposure intensity explained most of the rise in compound exposure across most regions, particularly in Asia (82.7%) and Oceania (81.8%), whereas population growth contributed relatively more in Africa (38.7%) than in other regions.

### Socio-economic disparities

Countries with high HDI scores and upper-middle-income countries accounted for the largest proportions of the global total exposed person-days over the period 2000–2019, representing ∼40% (3.8 billion person-days) and 49% (4.5 billion person-days) of the global total, respectively. However, low HDI and low-income countries exhibited the highest exposed days per person, with an overall average of 3.6 days and 4.4 days per person, respectively (Table 1).

Significant increases in population coexposure to heatwave and SFAP were observed across all socio-economic groups, except for high-income countries and those with very high HDI scores. Low HDI countries experienced the highest increase in exposed person-days, with an increase of 2.2 billion person-days/decade, followed by countries with high HDI, with an increase of 2.1 billion person-days/decade. Countries with low HDI also experienced the highest increase in exposed days per person, with an increase of 2.0 days per person per decade. Decomposition analysis further indicated that the relative contributions of population growth and exposure intensity differed across development levels. In low-HDI countries, population growth accounted for a larger share of the increase in coexposure person-days (40.6%) compared with 14.7% in very high-HDI countries. In contrast, increases in very high-HDI countries were primarily driven by rising coexposure intensity, which accounted for 85.3% of the total change (table S5). Similar results were observed across income groups (Fig. 4, Table 1, and table S5). Trends of three metrics for individual extreme events (heatwave or SFAP), stratified by socio-economic status, are shown in figs. S7 to S12.

### Leading countries in population coexposure to heatwave and SFAP

During the study period, the top five countries with the highest annual averages of person-days coexposure to heatwave and SFAP were: the Democratic Republic of the Congo, Indonesia, Mexico, Brazil, and the Russian Federation (fig. S13). These countries collectively accounted for over half (50.3%) of all exposed person-days in 2010–2019 (fig. S13). These countries were also those with the highest annual average numbers of persons and person-days exposed to either heatwave or SFAP (top-right corner of Fig. 5). The Democratic Republic of the Congo experienced the largest increase in person-days exposure, rising from 0.6 billion per year in 2000–2009 (ranked third) to 1.8 billion per year in 2010–2019 (ranked first), representing an increase of 1.1 billion person-days/decade (*P* for trend < 0.001) (fig. S13).

**Fig. 5.**
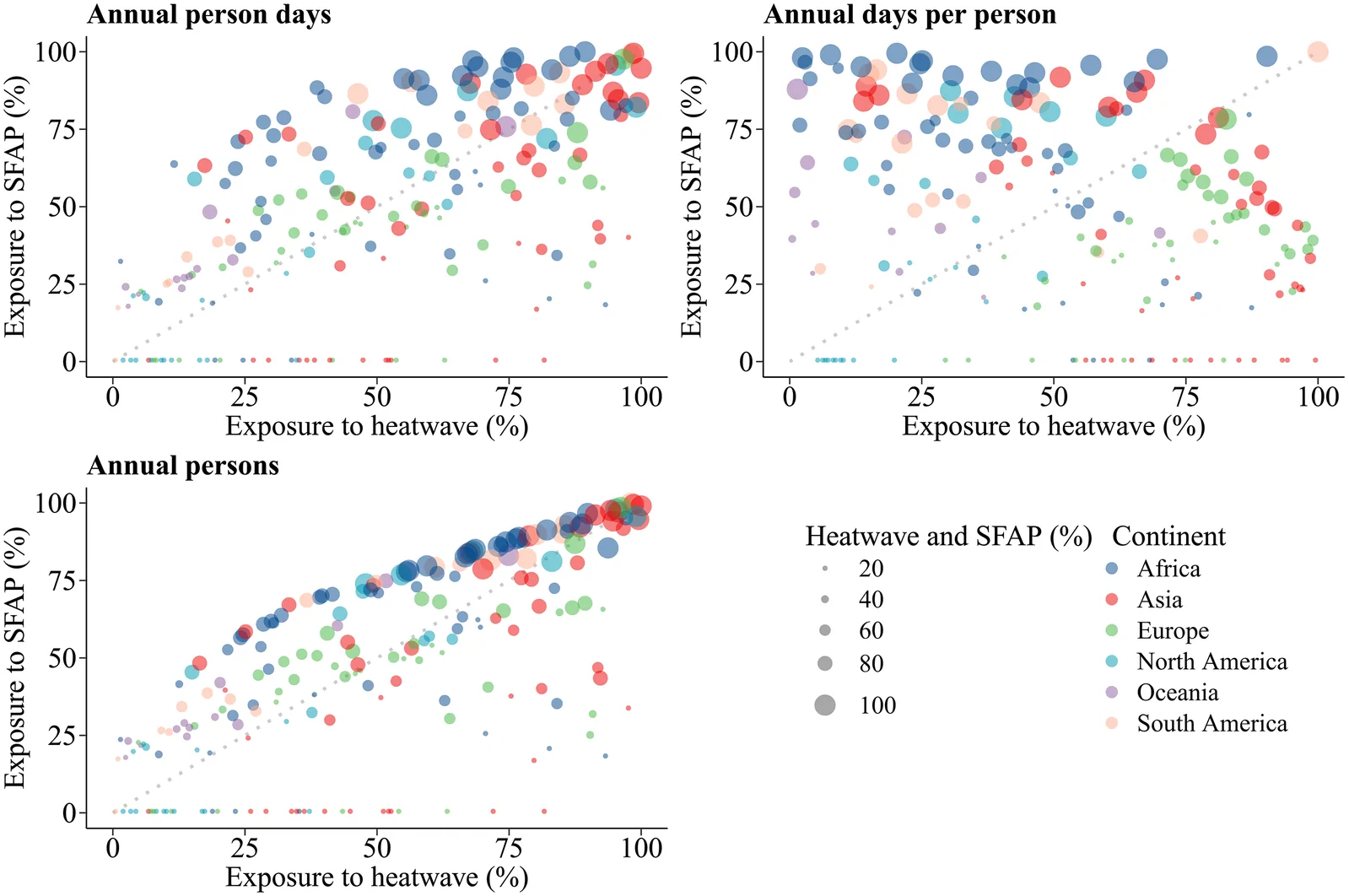
Scatter plot of countries based on percentiles of population exposure to heatwave, SFAP, and coexposure to heatwave and SFAP from 2000 to 2019. The *x* axis represents the percentile of exposure to heatwave, the *y* axis represents the percentile of exposure to SFAP, and the size of each dot corresponds to the percentile of coexposure to heatwave and SFAP.

After adjusting for population size, the leading countries with the highest annual days of coexposure per person were predominantly those with higher annual days of SFAP per person. However, these countries were not always the ones with the highest exposure to heatwaves (Fig. 5). For the period of 2010–2019, all the leading countries (top 10) for exposed days per person were low- and middle-HDI countries. The top five were: Zambia (28.8 days per person per year), the Democratic Republic of the Congo (24.3 days), Malawi (21.7 days), Guatemala (17.7 days), and Burundi (16.8 days). The Democratic Republic of the Congo showed the largest increase in exposed days per person, from 11.8 days per person in 2000–2009 (ranked fourth) to 24.3 days per person in 2010–2019 (ranked second) (*P* < 0.001 for trend, representing an increase of 11.4 days per person per decade). A notable shift occurred in Cambodia, which climbed from fortieth (1.6 days per person) in exposed days per person in 2000–2009 to ninth (12.1 days per person) in 2010–2019 (fig. S13).

### Sensitivity analyses

Sensitivity analyses using a more restrictive heatwave definition that combined relative and absolute temperature thresholds yielded consistent results, with only negligible differences in magnitude compared with the primary definition (table S6). In sensitivity analyses holding population constant at 2010 levels, total person-days and exposed population counts shifted in accordance with demographic adjustments, whereas coexposure days per person remained unchanged (table S7).

## DISCUSSION

This study provides a comprehensive assessment of global population coexposure to heatwave and SFAP over the first two decades of this century. Approximately 69% of countries experienced at least one coexposure day. The global exposed population increased from 761 million per year in 2000–2009 to more than 1 billion per year in 2010–2019, reflecting an alarming upward trend. On average, each person was exposed to 1.3 coexposure days per year. Substantial geographical disparities were observed, with the largest and most significant increase in exposed days per person noted in Africa and the Americas. Moreover, low HDI countries have experienced the highest exposure per person and the largest increase in it, indicating socio-economic disparities.

Our assessment confirmed the increasing trend of coexposure to heatwave and SFAP globally and highlighted the severity of this specific climate crisis. Although compound extreme climate events are being studied, most studies focus primarily on heat and drought events, with only a few studies looking at landscape fires and heatwave compound events (*3*, *28*–*30*, *33*). For example, Rosenthal *et al.* (*30*) observed that 68% of the land area of California and 42% of the population experienced days with both heatwaves and extreme smoke in 2020. In our study, we estimated that more than 1 billion people per year were exposed to at least one coexposure day globally from 2010 to 2019. These findings consistently confirmed that substantial populations around the world are exposed to concurrent landscape fire and heatwave events. In addition, our analysis revealed that from 2000 to 2019, regions including Central America, South America, Central and Eastern Africa, Southeast Asia, and parts of North America, Australia, and Russia experienced notable increases in the annual average number of coexposure days. This is also partly consistent with previous findings showing that the co-occurrence of forest fires and heatwaves was more likely in regions such as North America, Eastern Europe, and Russia (*33*). Building on previous research, this study integrates high-resolution landscape fire-sourced pollutant data with grid-level population data to provide an accurate estimate of population coexposure to these climate hazards.

The complex interplay among climate change, heatwaves, and landscape fires has profound implications at both global and local scales. Large-scale atmospheric circulation patterns, such as the El Niño Southern Oscillation (ENSO), influence interannual variability in heatwave characteristics (*34*). ENSO events can increase the frequency, persistence, and spatial extent of heatwaves, often causing them to travel longer distances and progress more slowly, especially in regions such as Southeast Asia and Australia (*34*–*36*). These changes are further amplified by global warming, which not only raises baseline temperatures but also alters precipitation patterns and moisture availability. At the local scale, soil moisture and heat fluxes are intimately linked (*37*). Dry soils enhance sensible heat flux, which in turn reinforces upper-level anticyclonic anomalies that further exacerbate heatwaves (*38*, *39*). This process creates a positive feedback loop: High temperatures accelerate soil desiccation and reduce evaporative cooling, leading to greater atmospheric instability and a heightened demand for evaporation. This increased demand further intensifies sensible heat flux and can even trigger the onset of additional heatwave events through the boundary layer (*40*).

In parallel, fire occurrence depends on a delicate balance between vegetation growth (responsible for fuel production) and the frequency of dry conditions that govern fuel moisture (*41*). Regions with intermediate moisture availability tend to experience the highest fire incidence. In these areas, conditions are dry enough on occasion to desiccate vegetation and promote flammability, yet still moist enough to support robust fuel growth. This balance is a key determinant of fire weather, which is characterized by high temperatures, low humidity, and strong winds (*42*). Heatwaves and landscape fire-conducive conditions are intrinsically linked. Elevated temperatures, increased vapor pressure deficit, and soil moisture deficits are critical drivers of fire weather, increasing the likelihood of ignition and rapid fire spread (*2*). Moreover, landscape fires themselves contribute to climate feedback by emitting carbon dioxide and other greenhouse gases that further warm the atmosphere, potentially raising both the frequency and intensity of subsequent heatwaves (*43*).

As suggested by our results, while several hotspots showed an increasing trend of coexposure days, the driving factors behind this trend may vary from region to region. For example, in Russia and Canada, the observed rise in coexposure days coincided with an increase in SFAP days. These countries, which span from the Arctic polar zone to the temperate zone, have seen record-setting forest landscape fires during summer, driven by factors such as lightning and anthropogenic climate change (*44*, *45*). Some big fires may last for weeks or months until a substantial rain event occurs (*46*). Forest fires in these areas release higher amounts of ambient pollutants than savannas and grassland but with a slower follow-up recovery (*47*). In contrast, the increase in coexposure days in South America, Central and Eastern Africa, and Southeast Asia was primarily associated with a rise in heatwave days. This pattern may result from the combined effects of a strong El Niño and human-induced climate change, while rapid urbanization and land-use change (e.g., deforestation, wetland loss), acting as long-term socio-land system transitions, may further contribute to increasing heat extremes through altered surface radiative and energy balances, including lower albedo, enhanced shortwave radiation absorption, and reduced evaporative cooling (*37*, *48*, *49*). The combination of high temperatures and a dry air mass can lead to an increase in vapor pressure deficit over an extended period, resulting in increased desiccation of fuels (*5*).

Globally, we identified a statistically significant increase in the number of coexposure person-days. The increase in coexposure person-days arose from two components: population growth, which increased the number of people potentially exposed to both hazards, and increasing coexposure days per person, which increased the exposure burden independently of population growth. Our decomposition analysis further showed that, at the global level, 70.5% of the increase in coexposure person-days was attributable to increasing coexposure intensity, whereas 29.5% was attributable to population growth (table S5). This finding suggests that the rise in coexposure person-days was primarily driven by increasing frequency of heatwave-SFAP events relative to population growth (i.e., coexposure days per person). However, while heatwave events followed a similar upward trend, SFAP remained relatively stable on a per-person basis, with no substantial increase in the annual days per person exposed. Therefore, the rise in the frequency of heatwave-SFAP events was primarily driven by more frequent heatwaves occurring during SFAP days, rather than by a global increase in SFAP frequency itself (*17*, *47*). Nevertheless, note that this represents a global-average pattern, and substantial regional heterogeneity exists. For example, we identified a pronounced increase in the annual number of SFAP days in western United States (fig. S3), consistent with the previous regional study (*2*). Correspondingly, North America is the only region where the annual number of SFAP exposure days per person increased over the study period (table S4). Furthermore, our analysis of the overlap between heatwave and SFAP revealed that the increase in person-days exposed to heatwave during SFAP events outpaced the rise in SFAP person-days during heatwave events. All these findings suggested that the rise in population coexposure to heatwave and SFAP was largely driven by the increasing intensity of heatwave, particularly on SFAP days.

The analysis highlights socio-economic disparities in population coexposure to heatwave and SFAP. We observed that low-income countries experienced the highest exposed days per person and the sharpest increase, which was more than three times that of higher socio-economic regions. This pattern emphasizes widening inequalities in climate-related vulnerabilities, with poorer nations disproportionately affected. The disparity may be partly attributed to several factors. Many low-income countries are located in tropical and subtropical regions where baseline temperatures are high, such that incremental warming more readily translates into frequent heat extremes. Rapid population growth further amplifies population exposure. These areas are also characterized by frequent agricultural waste burning and intentional deforestation for land use, along with limited landscape fire management (*17*). Moreover, this inequality extends beyond population exposure and also affects the health effects of coexposure to heatwave and landscape fire events. Previous studies indicated that communities with lower socio-economic status experience higher synergistic health effects from heatwaves and landscape fires (*4*). This disparity could be linked to various social determinants of health, including limited access to health care, reduced financial means to mitigate hazard exposure, lower awareness of combined environmental risks, and a higher prevalence of pre-existing health conditions (*4*). The amplification of disparities in both exposure and health effects further exacerbates the health burden of compound heatwave and landscape fire events in low-income countries.

Our findings have important implications. First, we show that global population coexposure to heatwave and SFAP are substantial and increasing substantially. These results provide a quantitative foundation for future epidemiological studies investigating the interactive health effects of heat and landscape fire smoke. It is therefore important to advance epidemiological research that considers compound exposures, including re-evaluating the minimum risk (or minimum mortality) temperature and reassessing human tolerance thresholds for heatwaves during periods when landscape fire smoke is also present. This research would inform the development of refined health risk thresholds and enhance predictive models for public health planning. Second, traditional risk management strategies designed to address single environmental hazards in isolation are insufficient for responding to compound events. Risk management frameworks must evolve to tackle the challenges posed by concurrent heatwaves and landscape fire smoke. This evolution includes developing coordinated emergency response plans that address both hazards, enhancing surveillance systems to monitor health outcomes associated with combined exposures and ensuring that health care infrastructure is resilient to these events. For example, given that simultaneous exposure could increase the risk of multiple adverse health effects (*4*), health care systems should adopt integrated approaches that address the full spectrum of impacts, including heat stress, respiratory distress, and cardiovascular complications. Third, adaptation strategies must focus on reducing population vulnerability to concurrent heatwaves and SFAP. A key priority is improving the capacity of individuals to shelter in place safely during these events. This includes providing accessible emergency shelters, particularly for vulnerable populations without adequate home protection, and ensuring that indoor environments offer both thermal comfort and protection from air pollutants. Long-term adaptation strategies should promote climate-resilient building designs that incorporate mechanical ventilation with particle filtration and gas capture capabilities while also being energy-efficient and powered by clean energy sources. Although these infrastructure investments are challenging, they are essential for reducing the health burden of compound climate hazards. Last, effective communication is crucial in mitigating the health risks associated with concurrent heatwaves and landscape fires. A recent review observed that only a small subset of public health agency messaging was designed to address coexposure to landscape fire smoke and heatwave, with the majority focusing on individual hazards without fully addressing their co-occurrence (*50*). This highlights the need for more precise and coordinated public health messaging with clear and actionable guidance to protect against health outcomes attributable to concurrent heatwave and landscape fire events.

This study has several strengths. This study offers the largest global assessment of population coexposure to heatwaves and landscape fire smoke, providing comprehensive spatial and temporal insights across all populated continents. By covering a two-decade period (2000–2019), the study captures long-term trends and allows for a robust evaluation of temporal dynamics and emerging hotspots of coexposure. The use of high-resolution gridded data enables detailed sub-national analyses, enhancing the precision of exposure assessment and supporting localized public health planning. In addition, we extend beyond same-day co-occurrence by incorporating a superposed epoch analysis to characterize the temporal sequencing between heatwaves and smoke events. This enables evaluation of whether landscape fires tend to precede, coincide with, or follow heatwave onset, providing deeper insight into compound hazard dynamics. The integration of sociodemographic data allows for the assessment of exposure disparities across countries with different human development levels, revealing critical environmental justice dimensions.

The limitations of this study are primarily due to uncertainties in estimating landscape fire-sourced PM_2.5_ and O_3_ (*17*). Fire emissions create a complex and dynamic mixture of pollutants that vary across time and space. The GEOS-Chem model, while robust, does not fully account for fire plume rise, potentially leading to overestimations of fire emission contributions to surface PM_2.5_ and O_3_ in source regions and underestimations in downwind areas (*51*). The spatial resolution of the GEOS-Chem outputs is relatively coarse, potentially underestimating the spatial variability of exposure. Although we downscaled daily total and landscape fire-sourced PM_2.5_ and O_3_ to a finer resolution of 0.25° × 0.25°, this process may have introduced some degree of exposure misclassification. In addition, carbon monoxide (CO), another notable landscape fire pollutant, was not considered in our models. However, since CO impacts are typically localized near fire sources, its exclusion was unlikely to substantially affect our estimates of population exposure to landscape fire-related air pollution (*52*). Last, our analysis focuses on population coexposure to heatwave and SFAP and does not explore the underlying land-atmosphere mechanisms (e.g., soil moisture anomalies, antecedent precipitation deficits) that give rise to these events. Future studies integrating exposure metrics with mechanistic land-atmosphere diagnostics will be essential to further elucidate the drivers of compound events.

In conclusion, our study highlights the severity and socioeconomic disparity in rising global population coexposure to heatwave and SFAP and provides high-resolution data to inform health impact assessments and targeted mitigation and adaptation strategies. As climate change continues to drive the frequency and intensity of these events, future research should focus on understanding their health impacts and developing comprehensive mitigation frameworks.

## MATERIALS AND METHODS

### Data source

#### Meteorological data

We obtained gridded hourly temperature data from the fifth-generation European Centre for Medium-Range Weather Forecasts Reanalysis (ERA5) dataset, at a resolution of 0.25° × 0.25° (*53*). The gridded hourly data were aggregated to compute daily averages for each grid cell based on the local time zone.

#### Landscape fire-sourced air pollution data

We used global daily landscape fire-sourced air pollution (PM_2.5_ and O_3_) data from 2000 to 2019 at a spatial resolution of 0.25° × 0.25° (∼28 km by 28 km), as detailed in our previous work (*17*). Briefly, we obtained fire emissions from the Global Fire Emissions Database (v4.1 with small fires), which integrates satellite-based burned area and active fire detections across major fire types including boreal forest fires, tropical forest fires, temperate forest fires, savanna, grassland and shrubland fires, peatland fires, and agricultural waste burning. We estimated daily global concentrations of total (all-source) PM_2.5_ and O_3_ using the GEOS-Chem chemical transport model (version 12.0.0), which simulates coupled O_3_-NO*_x_*-hydrocarbon-aerosol chemistry at a 2.0° × 2.5° horizontal resolution. Landscape fire-sourced PM_2.5_ and O_3_ concentrations were quantified by conducting paired simulations with and without landscape fire emissions and calculating the difference between the two model outputs.

We downscaled daily total and fire-attributable PM_2.5_ and O_3_ produced by GEOS-Chem from the native model resolution to 0.25° × 0.25° using inverse distance weighting interpolation. The downscaled pollutant fields were calibrated against ground-based air quality monitoring observations using a random forest machine-learning algorithm. This calibration reduced systematic bias in the chemical transport model outputs and improved agreement with observed surface concentrations. Final landscape fire-sourced PM_2.5_ and O_3_ concentrations were obtained by scaling the downscaled GEOS-Chem fire contributions. These contributions were scaled by the ratio of machine-learning-estimated total concentrations to GEOS-Chem total concentrations for each grid cell and day. The models demonstrated high predictive accuracy, with 10-fold cross-validation *R*^2^ values of 0.91 for PM_2.5_ and 0.82 for O_3_. Validation against station-based smoke PM_2.5_ estimates and observations from major landscape fire events indicated strong correlations (*R*^2^ = 0.64 and 0.78 for PM_2.5_ and O_3_).

#### Population and socioeconomic data

We collected annual population data at 30 arc sec (∼1 km^2^) spatial resolution from the WorldPop project for the years 2000–2019 (*54*). We then aggregated these gridded population counts to a 0.25° × 0.25° resolution to align with the air pollution and meteorological data. To ensure consistency with United Nations (UN) population data, we adjusted the population counts for each country by multiplying grid-specific counts within its boundaries by an adjustment coefficient, calculated as the ratio of the UN-reported population size to the sum of all grid-specific population counts within the boundary.

We downloaded gross national income (GNI) per capita data from the World Bank (https://data.worldbank.org/). Countries were classified according to the World Bank’s 2019 income criteria into four categories: low-income (GNI per capita ≤ US$1035), lower-middle–income countries (US$1035 < GNI per capita ≤ US$4045), upper-middle–income countries (US$4045 < GNI per capita ≤ US$12,535), and high-income countries (GNI per capita > US$12,535). We downloaded the 2019 HDI data from the United Nations Development Programme (UNDP). HDI was categorized according to the UNDP into four levels: very high (≥0.8), high (0.700 to 0.799), medium (0.550–0.699), and low human development (<0.550).

### Statistical analysis

#### Definition of coexposure to heatwave and SFAP

For each grid cell, consistent with our previous study, we defined a SFAP day as any day that met at least one of the following two criteria: (i) the daily average all-source PM_2.5_ exceeded the 2021 WHO daily guideline value of 15 μg/m^3^, with landscape fire-sourced PM_2.5_ contributing at least 50% of the all-source PM_2.5_. (ii) The daily maximum 8-hour all-source O_3_ exceeded the 2021 WHO daily guideline value of 100 μg/m^3^, with landscape fire-sourced O_3_ contributing at least 50% of the total O_3_ (*17*). Heatwave days were identified in each grid cell based on the daily mean temperature over 2000–2019 in that cell. There are many different definitions of heatwaves, including days with daily mean temperature exceeding a certain threshold (90th, 92.5th, 95th, or 97.5th percentile of daily mean temperature over the selected period) for a consecutive period (2 to 4 days) (*55*). We used the most lenient definition of heatwave (i.e., exceeding the 90th percentile for at least two consecutive days) to capture as many heatwave days as possible. Coexposure days were defined as days when SFAP and heatwaves occurred on the same day.

#### Temporal sequencing of heatwaves and SFAP

To quantify the temporal alignment between heatwave and SFAP events, we conducted a superposed epoch analysis (SEA) at the global grid-cell level (*56*, *57*). Heatwave onset days were defined as the first day of each heatwave episode, preceded by a non-event day. To ensure independence between episodes, successive onset events were required to be separated by at least 14 days. Grids were retained only if they experienced at least five independent heatwave onsets and five smoke onsets during the study period. For each anchor onset (heatwave), we extracted a symmetric ±7-day window and recorded whether an SFAP onset occurred on each relative day within this window. For each grid cell, SEA curves were constructed by calculating the mean probability of SFAP onset at each relative lag day across all valid heatwave onset events.

To assess whether the observed temporal alignment exceeded random expectation, we implemented a month-constrained permutation procedure. For each grid, we randomly sampled pseudo-onset days within the same calendar month as observed onsets, subject to the same 14-day minimum separation rule, and the SEA procedure was repeated (*K* = 30 permutations per grid). We then aggregated grid-level observed and pseudo-SEA curves to the global level using weights proportional to population size multiplied by the number of anchor events in each grid. To derive uncertainty intervals, one pseudo-SEA trajectory per grid was randomly drawn and population-weighted aggregation was repeated 1000 times to construct a global null distribution. The 2.5th and 97.5th percentiles of the null distribution were used as confidence bounds. We further conducted stratified analyses by continent using the same weighting and resampling framework.

#### Calculation of local temperature anomalies

To evaluate whether SFAP events occur under anomalously warm conditions independent of regional climatic differences, we calculated local temperature anomalies and compared anomalies between SFAP and non-SFAP days. Daily temperature anomalies were calculated separately for each grid cell to account for spatial heterogeneity in baseline climate. For each grid cell, a local climatology was defined as the mean temperature for each day of the year (DOY) across the study period (2000–2019). The temperature anomaly on day t was then calculated as

$$A_{i,t}=T_{i,t}-{\bar{T}}_{i,\text{DOY}}$$

where $T_{i,t}$ represents the observed daily temperature at grid cell i on day t and ${\bar{T}}_{i,\text{DOY}}$ denotes the long-term mean temperature for the corresponding DOY at the same grid cell. This approach removes large-scale climatic gradients and enables comparisons across regions with substantially different temperature regimes.

Temperature anomalies on SFAP days were compared with matched non-SFAP control days within the same grid cell. For each SFAP day, control days were selected from days without SFAP occurrence falling within ±3 calendar days of the same DOY. This matching minimizes confounding arising from intra-annual temperature variation while preserving sufficient sample size for comparison. For each SFAP day, the mean temperature anomaly of the matched non-SFAP control days was calculated, and the difference between the SFAP-day anomaly and the matched control anomaly was obtained. Differences in temperature anomalies between SFAP and matched non-SFAP days were first calculated at the grid level and subsequently aggregated to regional and global scales by averaging grid-level results within geographic regions.

#### Population coexposure to heatwave and SFAP

We explored the overlap between heatwave and SFAP using two metrics: the proportion of SFAP days that coincided with heatwaves and the proportion of heatwave days that coincided with SFAP. By overlaying exposure data with year-specific gridded population distributions, we quantified the population coexposure to heatwave and SFAP from 2000 to 2019 using three complementary metrics: (i) Annual person-days of coexposure were calculated as the product of number of coexposure days and population in a given year at each grid cell, capturing the aggregate population burden of compound exposure; (ii) annual coexposure days per person were calculated as the average number of coexposure days experienced by each person in a year, thereby accounting for population size and isolating changes in exposure intensity; (iii) annual number of exposed people was defined as the number of populations experiencing at least 1 day of coexposure in a given year, indicating the population reach of compound events.

To identify the drivers of changes in population coexposure, we decomposed annual coexposure person-days into contributions from population change and coexposure intensity. Using 2000 as the baseline year, changes in person-days relative to baseline were decomposed into population-driven and exposure-driven components using Das Gupta decomposition analysis (*58*, *59*). Grid-level contributions were then aggregated to regional and global scales.

#### Analysis of disparities in population coexposure

To explore disparities in population coexposure to heatwave and SFAP, we aggregated three metrics above by continent and socio-economic groups (income level and HDI score). We then determined the decadal change in three metrics of population coexposure to heatwave and SFAP. A linear regression model, assuming a Gaussian distribution of each metric on the log scale, was applied. Change per decade was calculated by multiplying the slope of the linear trend by ten. We calculated the *P* value for trends to test the significance of temporal trends.

#### Sensitivity analysis

To evaluate the robustness of our findings to the choice of heatwave definition and to address concerns that relative thresholds may identify heatwaves occurring at low absolute temperatures in high-latitude regions, we conducted sensitivity analyses using a more restrictive definition of heatwaves, requiring at least two consecutive days with daily mean temperature exceeding the local 95th percentile and an absolute temperature threshold of 15°C. In addition, we conducted a sensitivity analysis in which population was held constant at 2010 levels to isolate climate-driven changes in exposure independent of population growth and redistribution.
