# Supplementary material for: Rising global population coexposure to heatwaves and landscape fire-sourced air pollution
Source: Sci Adv. 2026 Oct 14;12(42):eaea9749. doi: 10.1126/sciadv.aea9749 (PMC13666011; doi:10.1126/sciadv.aea9749)
Supplement: Supplementary file 1 [file sciadv.aea9749_sm.pdf]

Science Advances
AAAS

Supplementary Materials for

Rising global population coexposure to heatwaves and landscape fire-sourced air pollution

Yao Wu *et al.*

Corresponding author: Shanshan Li, shanshan.li@monash.edu; Yuming Guo, yuming.guo@monash.edu

Sci. Adv. **12**, eaea9749 (2026)
DOI: 10.1126/sciadv.aea9749

This PDF file includes:

Figs. S1 to S13
Tables S1 to S7

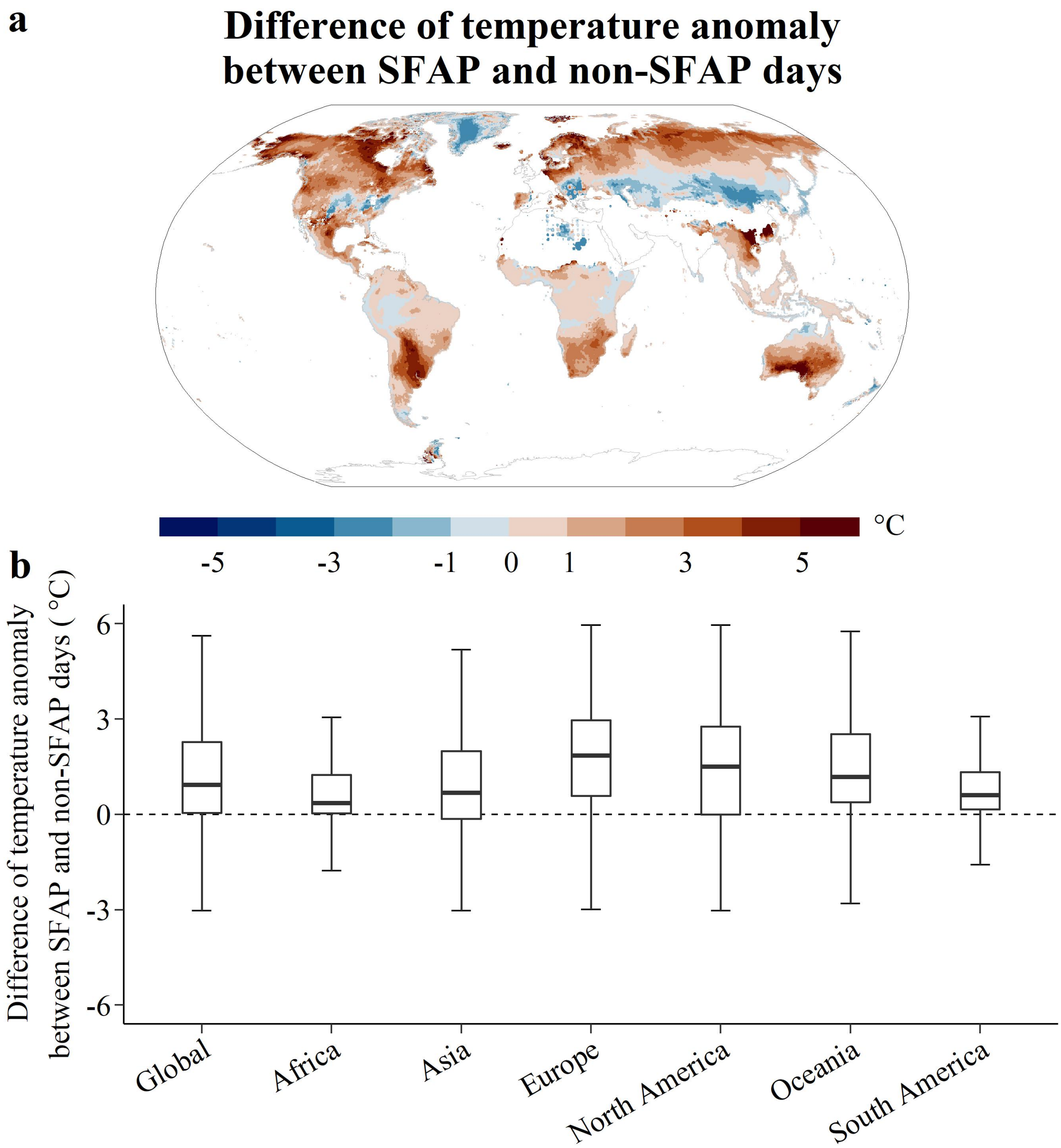

Fig. S1.

Differences in local temperature anomalies between SFAP and non-SFAP days. (a) Global spatial distribution of the mean difference in local daily temperature anomalies between SFAP and matched non-SFAP control days (°C). (b) Regional distributions of grid-level differences in local temperature anomalies between SFAP and matched non-SFAP days. Box plots show the median (centre line), interquartile range (box), and 1.5 times interquartile range (whiskers). The dashed horizontal line denotes zero difference.

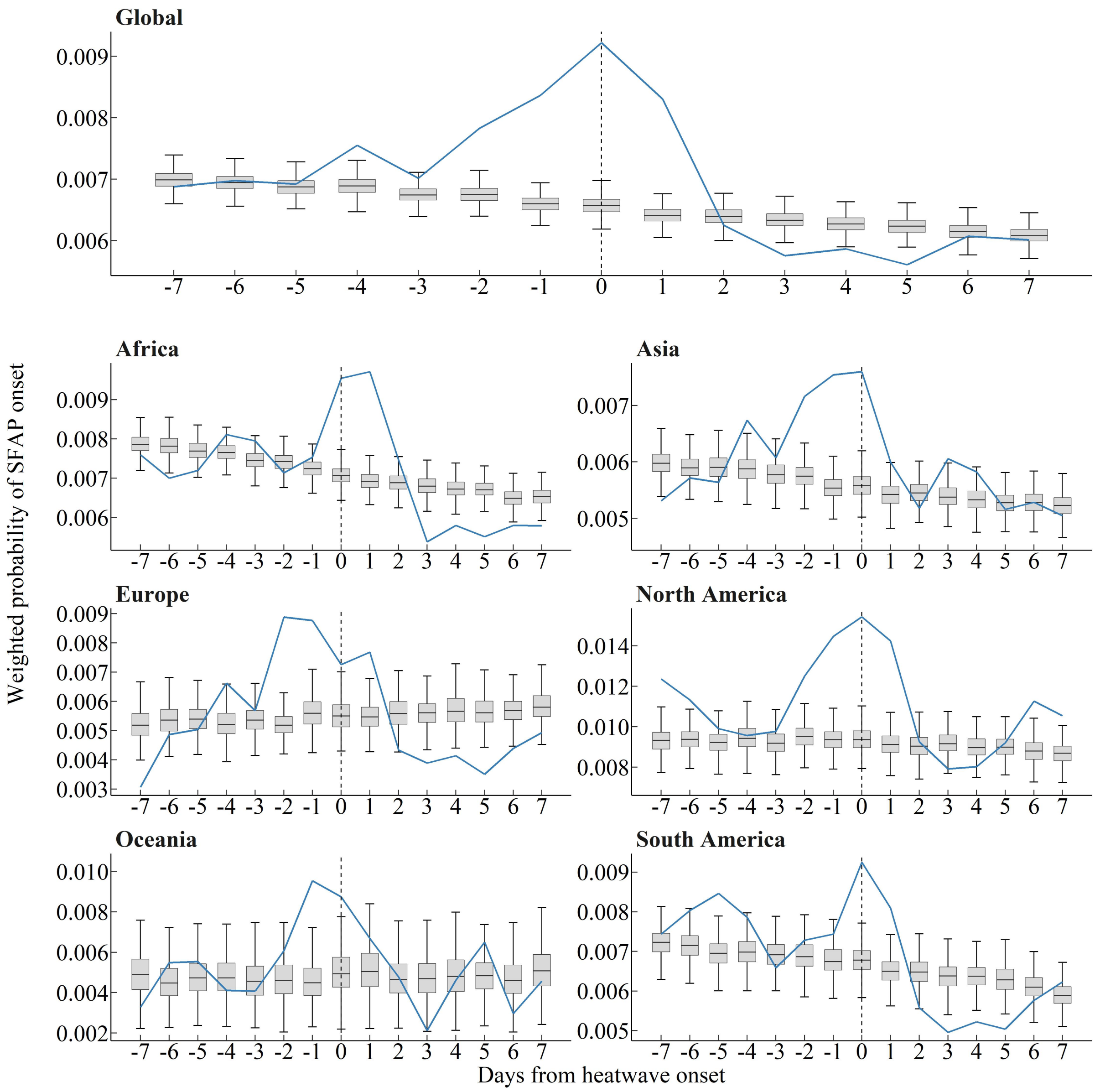

Fig. S2.

Temporal sequencing between heatwave onset and landscape fire-sourced air pollution (SFAP). Blue lines represent the superposed epoch composites of SFAP onset probability from 7 days before to 7 days after heatwave onset. Grey boxes represent the baseline distribution of SFAP onset probabilities for the corresponding calendar days across the study period, independent of heatwave timing.

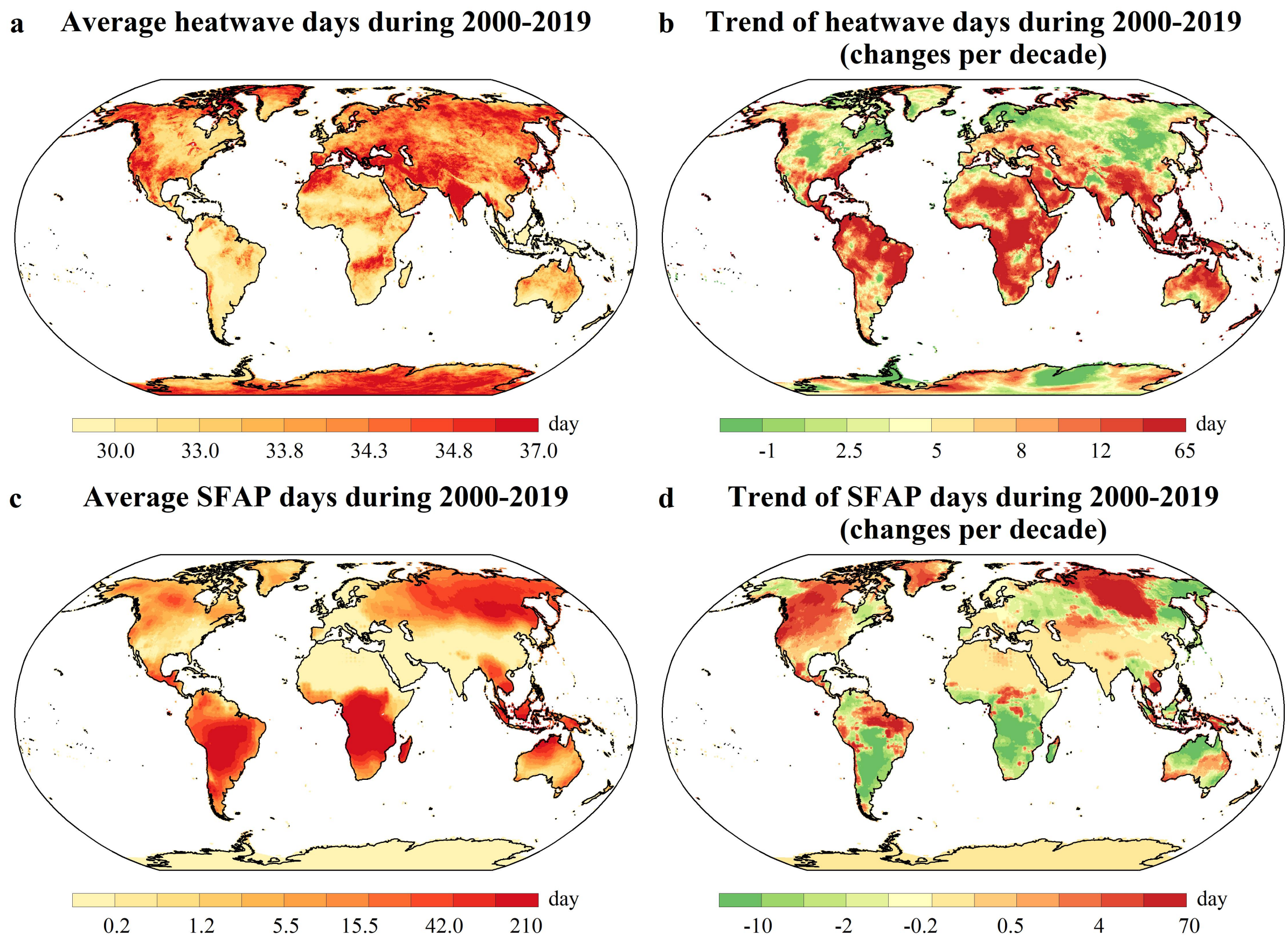

Fig. S3.

Global maps of annual average heatwave and substantial fire-sourced air pollution (SFAP) days during 2000–2019 and the estimated trend during the period. (a) annual average heatwave days; (b) trend in heatwave days (change per decade); (c) annual average SFAP days; and (d) trend in SFAP days (change per decade). The trend from 2000 to 2019 was fitted using all annul values during the period with a linear regression.

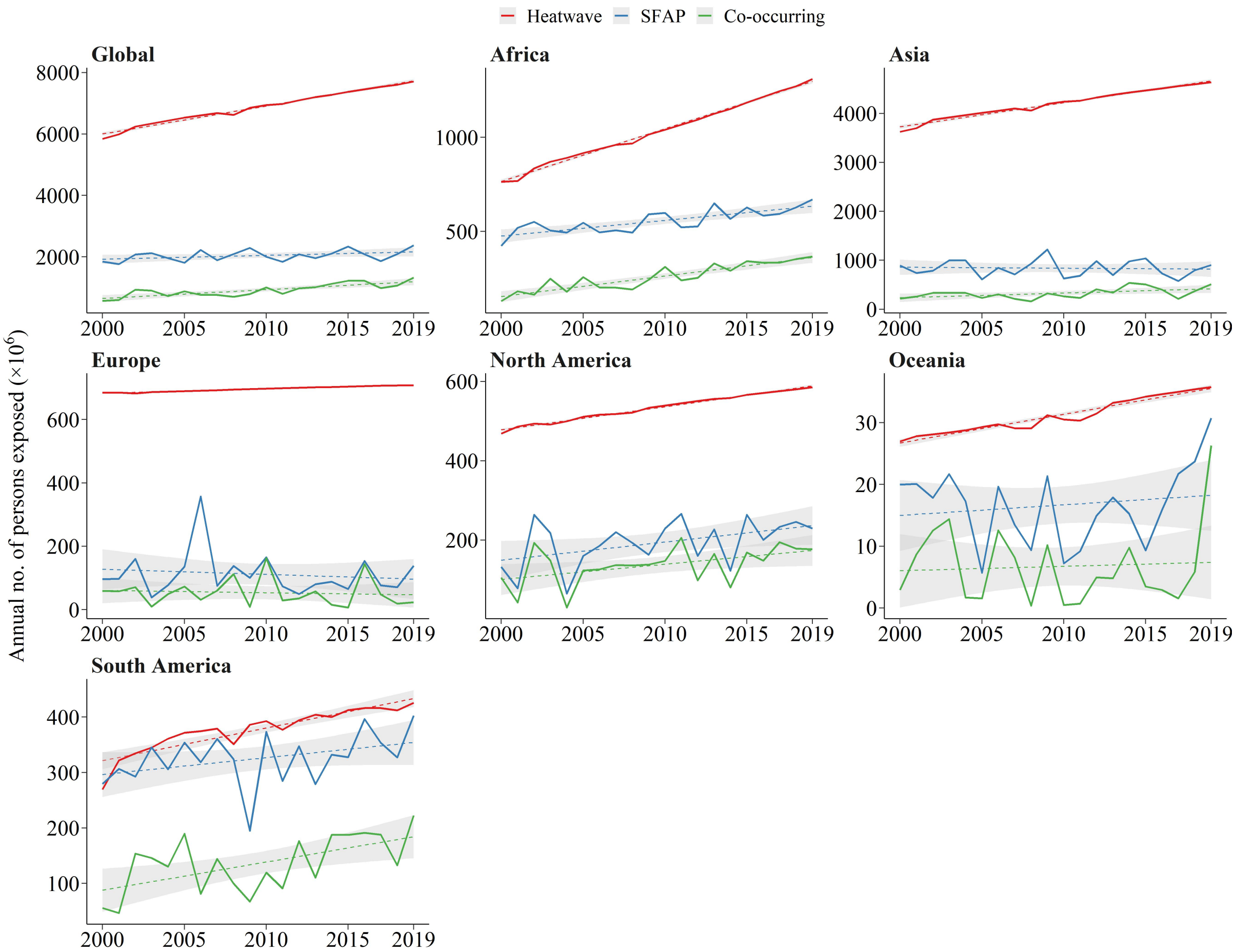

Fig. S4.

Annual number of persons exposed to heatwave (red lines), substantial fire-sourced air pollution (SFAP) (blue lines), and co-exposure to heatwave and SFAP (green lines) from 2000 to 2019 by continent. The dashed lines denote point estimates of the fitted trend by linear regression and the shaded areas denote the corresponding 95% confidence intervals.

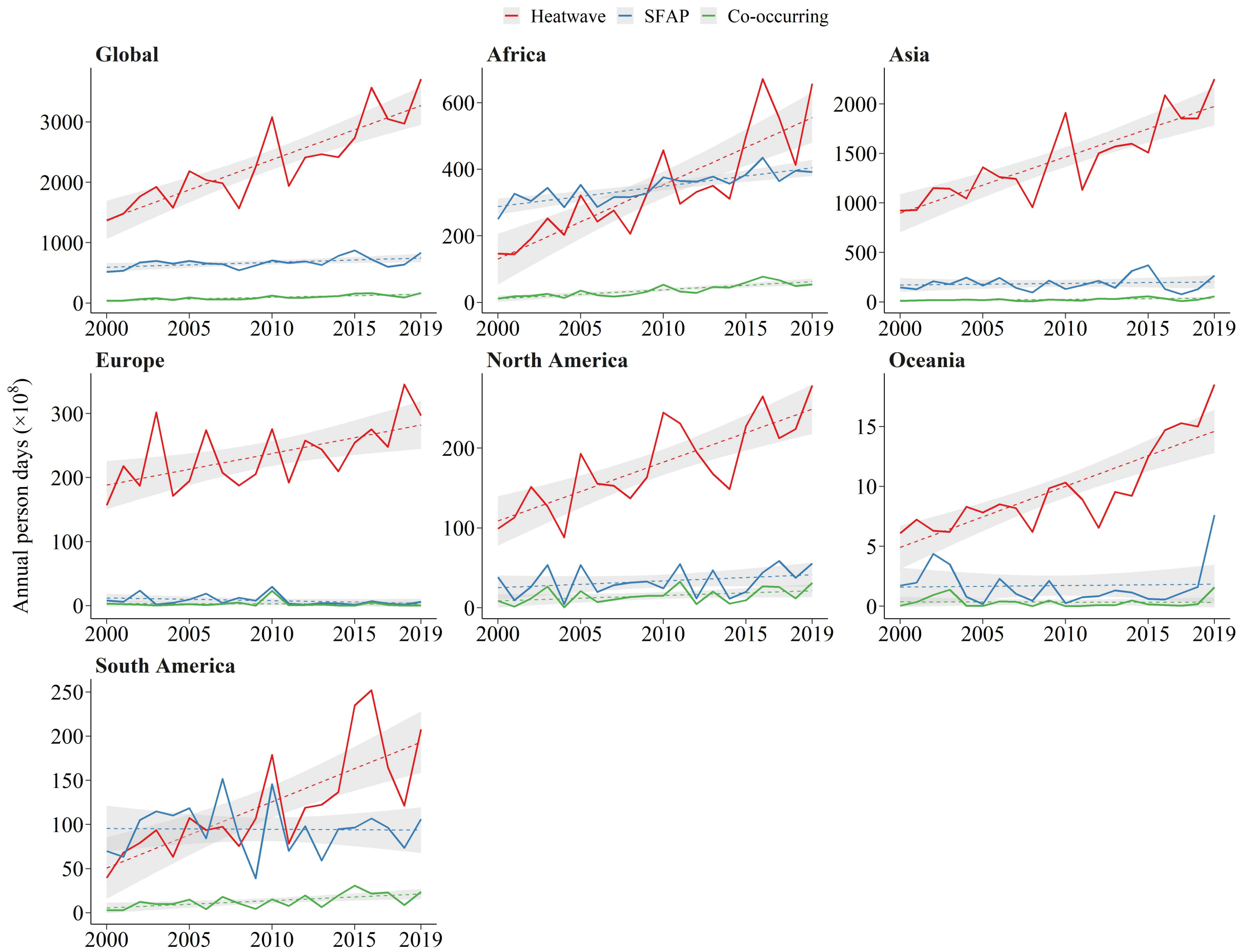

Fig. S5.

Annual person-days exposed to heatwave (red lines), substantial fire-sourced air pollution (SFAP) (blue lines), and co-exposure to heatwave and SFAP (green lines) from 2000 to 2019 by continent. The dashed lines denote point estimates of the fitted trend by linear regression and the shaded areas denote the corresponding 95% confidence intervals.

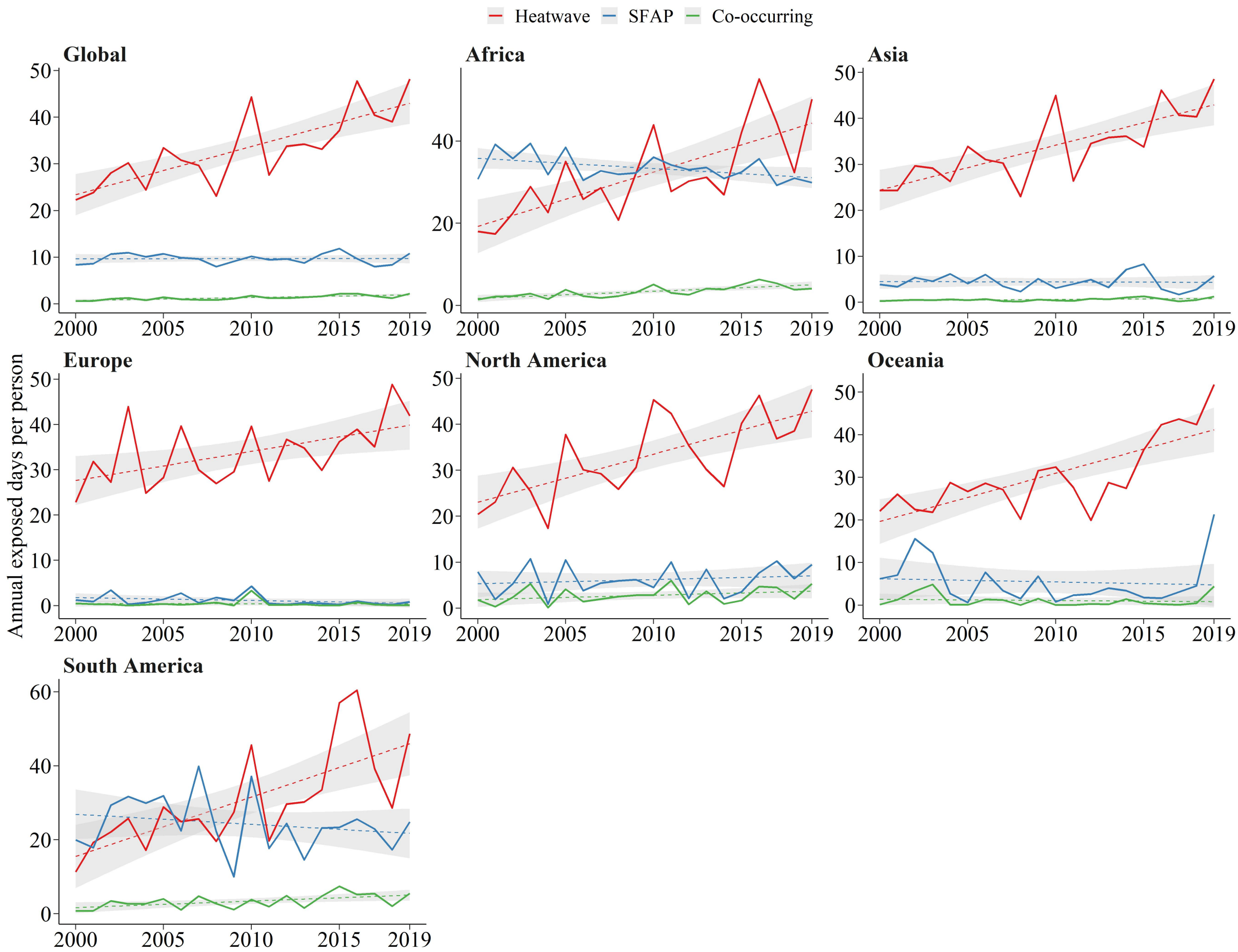

Fig. S6.

Annual days per person exposed to heatwave (red lines), substantial fire-sourced air pollution (SFAP) (blue lines), and co-exposure to heatwave and SFAP (green lines) from 2000 to 2019 by continent. The dashed lines denote point estimates of the fitted trend by linear regression and the shaded areas denote the corresponding 95% confidence intervals.

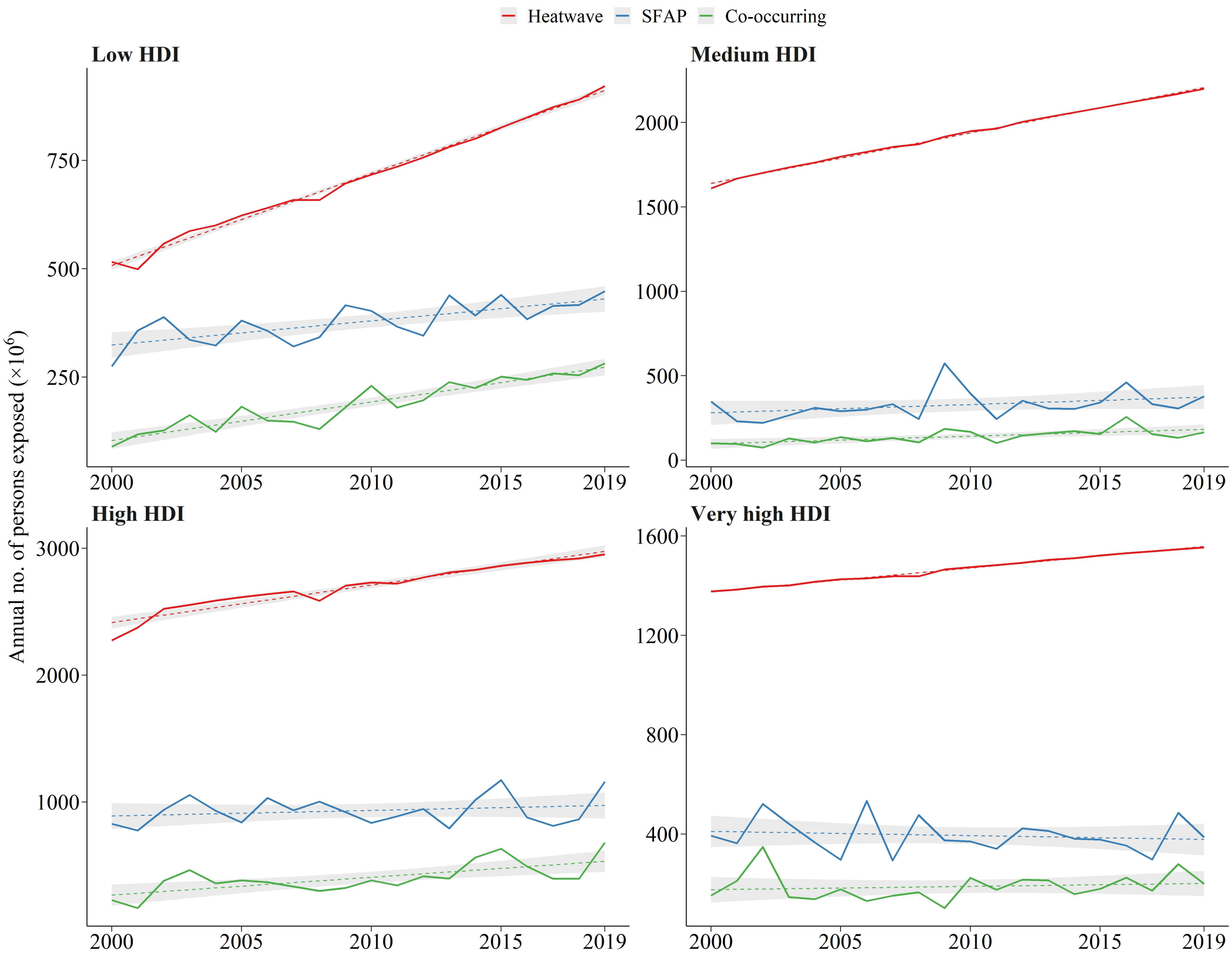

Fig. S7.

Annual number of persons exposed to heatwave (red lines), substantial fire-sourced air pollution (SFAP) (blue lines), and co-exposure to heatwave and SFAP (green lines) from 2000 to 2019 by human development index (HDI) level. The dashed lines denote point estimates of the fitted trend by linear regression and the shaded areas denote the corresponding 95% confidence intervals.

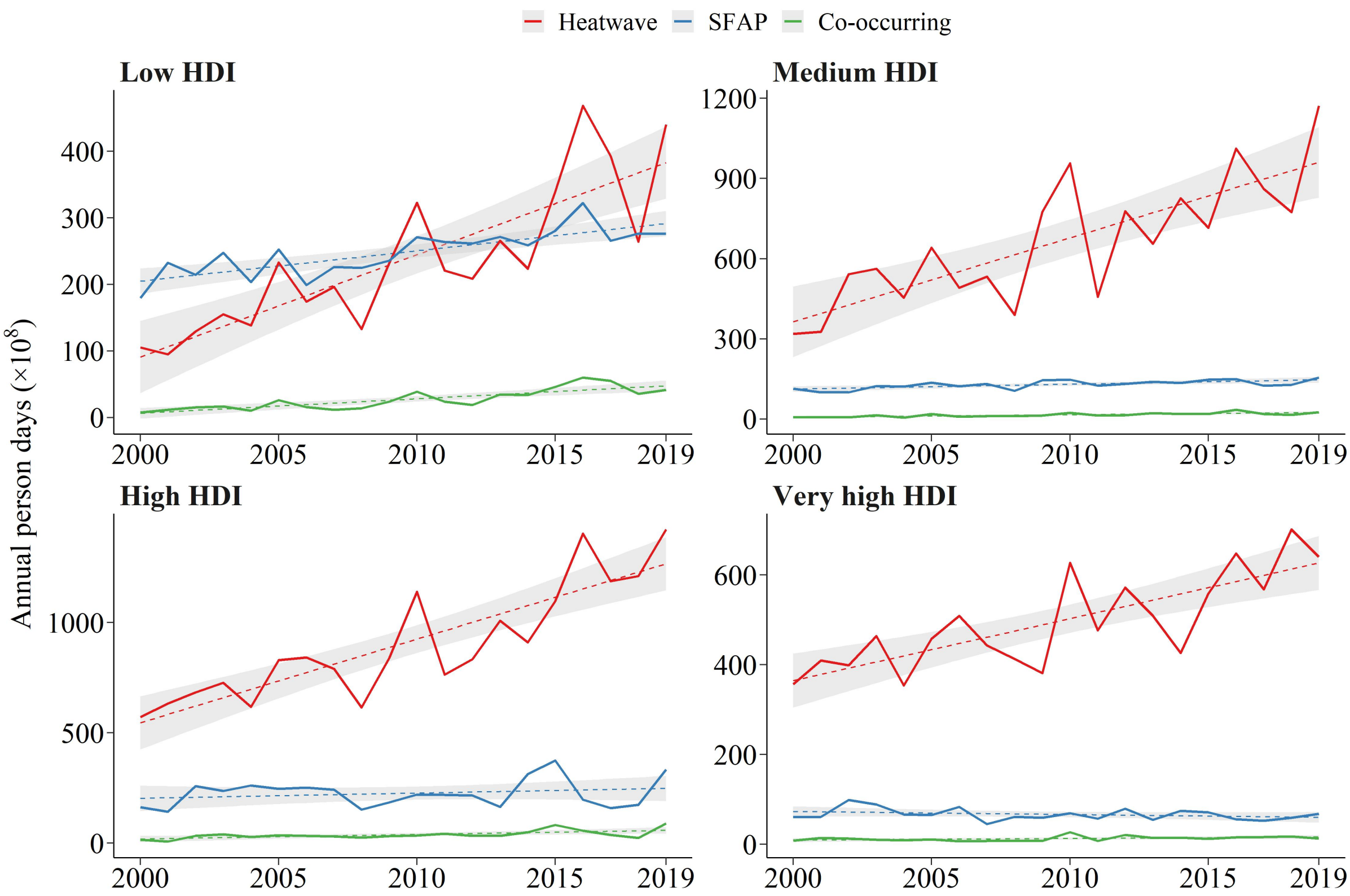

Fig. S8.

Annual person-days exposed to heatwave (red lines), substantial fire-sourced air pollution (SFAP) (blue lines), and co-exposure to heatwave and SFAP (green lines) from 2000 to 2019 by human development index (HDI) level. The dashed lines denote point estimates of the fitted trend by linear regression and the shaded areas denote the corresponding 95% confidence intervals.

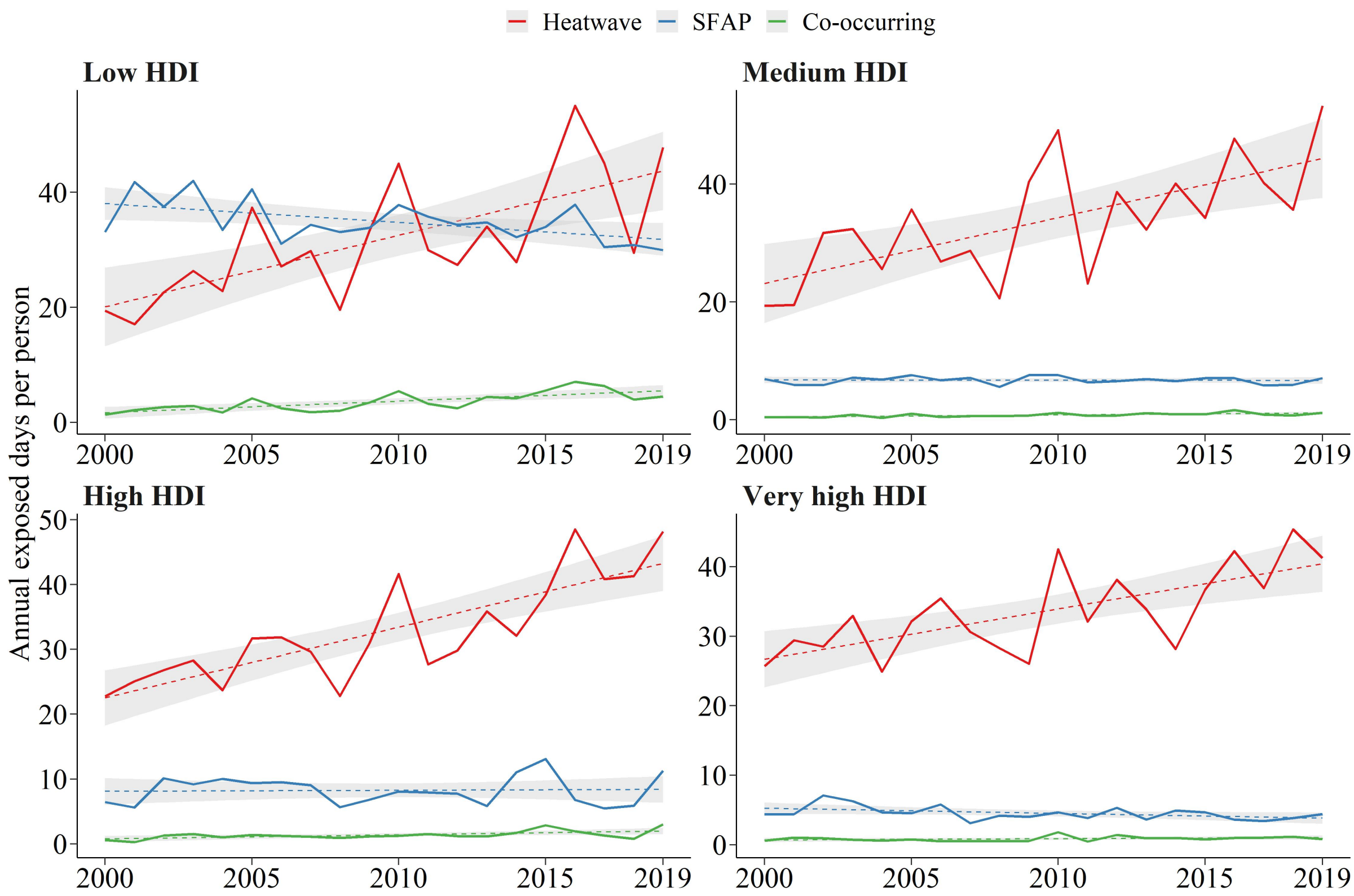

Fig. S9.

Annual days per person exposed to heatwave (red lines), substantial fire-sourced air pollution (SFAP) (blue lines), and co-exposure to heatwave and SFAP (green lines) from 2000 to 2019 by human development index (HDI) level. The dashed lines denote point estimates of the fitted trend by linear regression and the shaded areas denote the corresponding 95% confidence intervals.

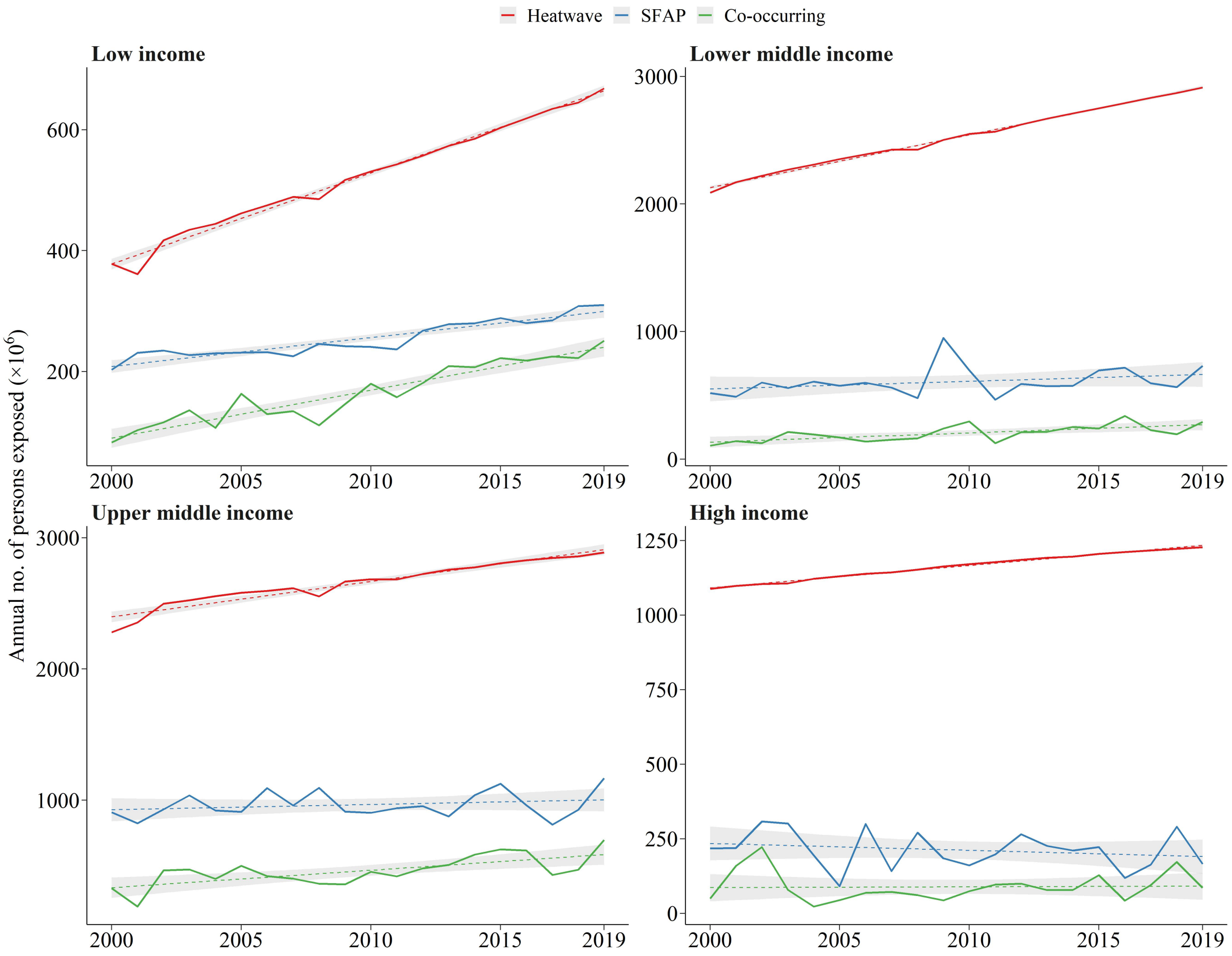

Fig. S10.

Annual number of persons exposed to heatwave (red lines), substantial fire-sourced air pollution (SFAP) (blue lines), and co-exposure to heatwave and SFAP (green lines) from 2000 to 2019 by income level. The dashed lines denote point estimates of the fitted trend by linear regression and the shaded areas denote the corresponding 95% confidence intervals.

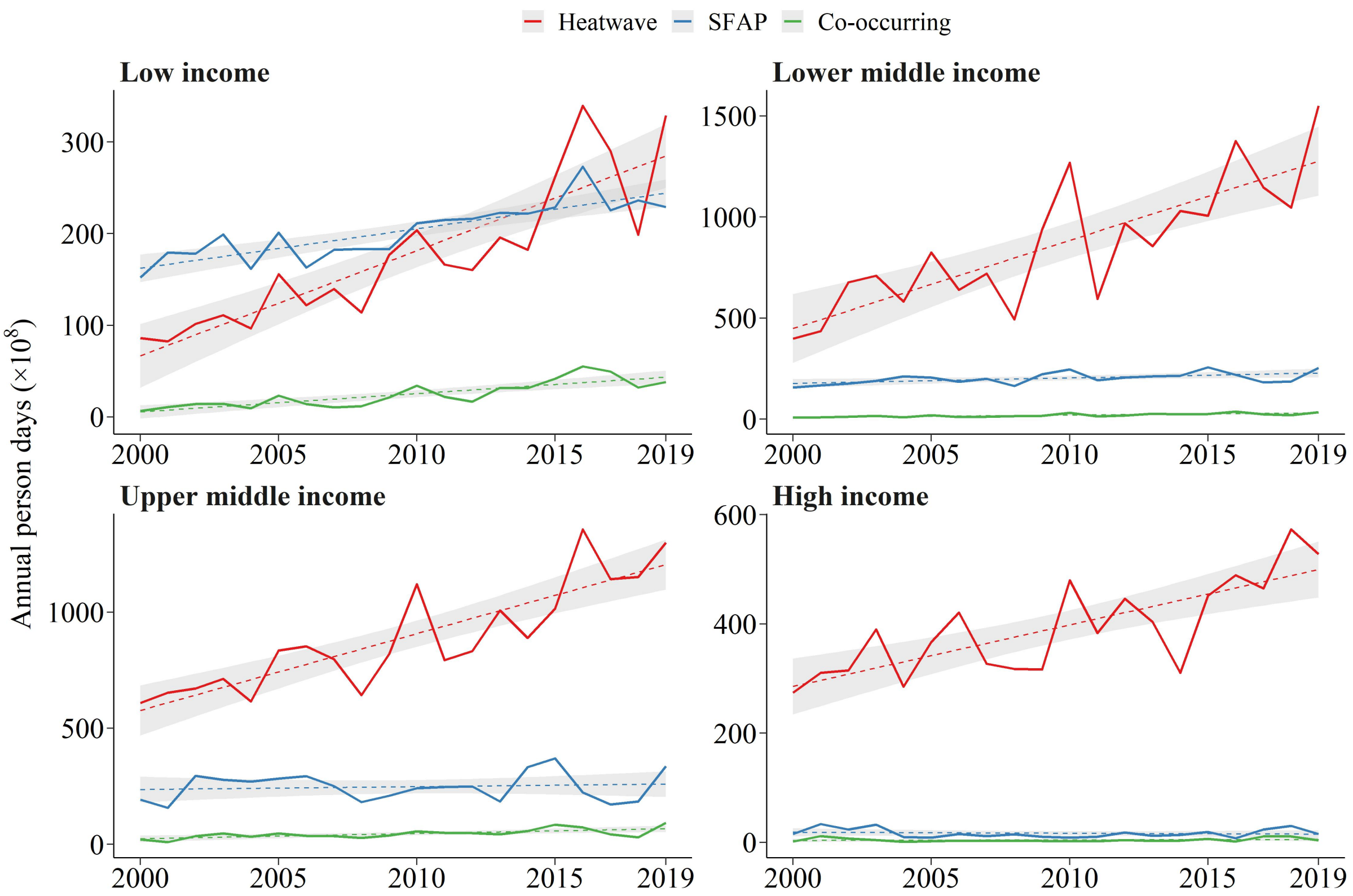

Fig. S11.

Annual person-days exposed to heatwave (red lines), substantial fire-sourced air pollution (SFAP) (blue lines), and co-exposure to heatwave and SFAP (green lines) from 2000 to 2019 by income level. The dashed lines denote point estimates of the fitted trend by linear regression and the shaded areas denote the corresponding 95% confidence intervals.

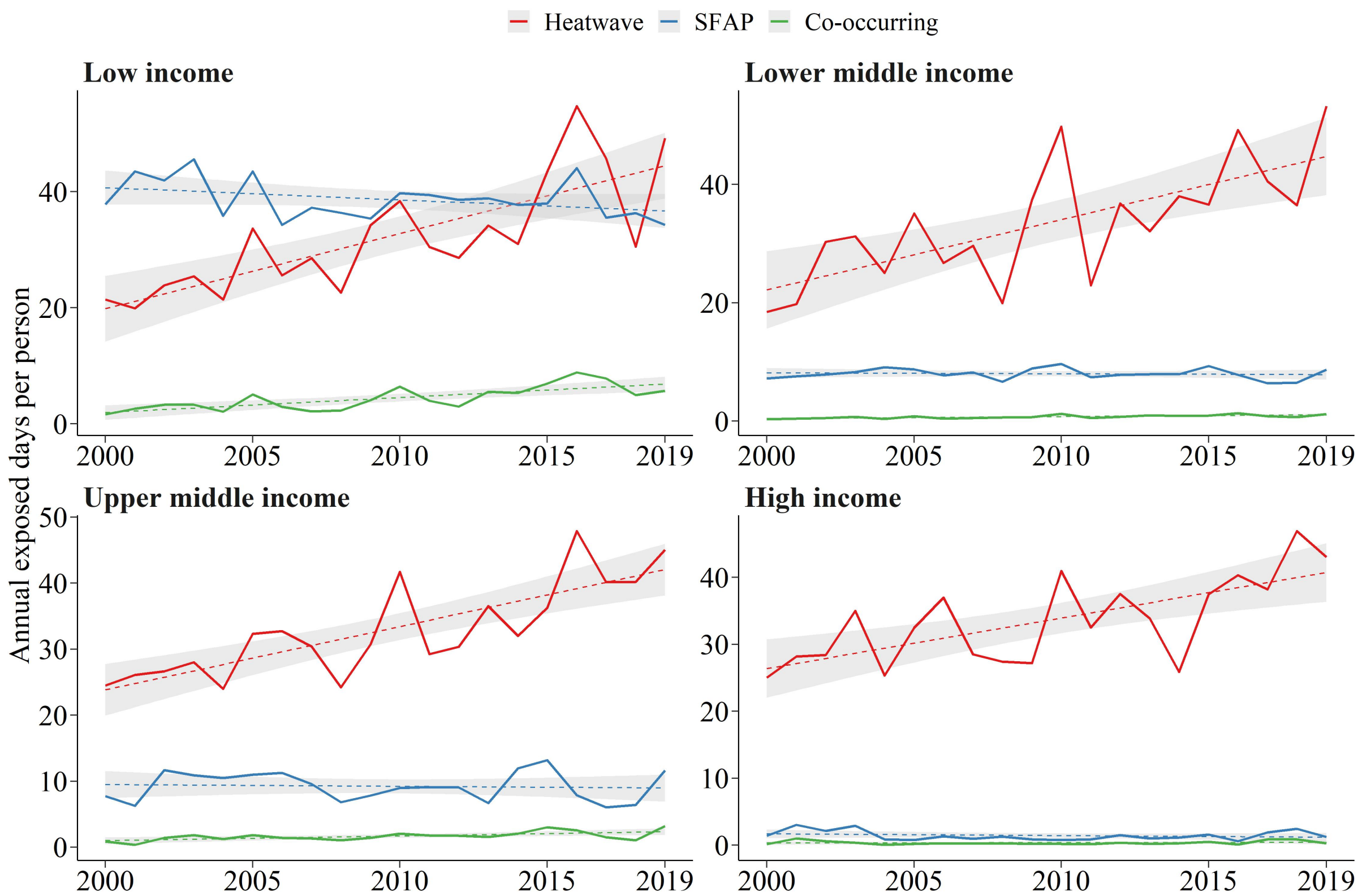

Fig. S12.

Annual days per person exposed to heatwave (red lines), substantial fire-sourced air pollution (SFAP) (blue lines), and co-exposure to heatwave and SFAP (green lines) from 2000 to 2019 by income level. The dashed lines denote point estimates of the fitted trend by linear regression and the shaded areas denote the corresponding 95% confidence intervals.

a

Country	Exposed person days in 2000-09 (%)	Country	Exposed person days in 2010-19 (%)
1 Mexico	0.8 (13.1)	1 Democratic Republic of the Congo	1.8 (15.0)
2 Indonesia	0.8 (12.4)	2 Indonesia	1.3 (10.7)
3 Democratic Republic of the Congo	0.6 (10.3)	3 Brazil	1.2 (9.5)
4 Brazil	0.6 (9.5)	4 Mexico	1.2 (9.4)
5 Russian Federation	0.3 (5.1)	5 Russian Federation	0.7 (5.7)
6 Zambia	0.3 (4.1)	6 Zambia	0.5 (3.7)
7 Angola	0.2 (3.6)	7 Angola	0.4 (3.5)
8 Malawi	0.2 (2.8)	8 Malawi	0.4 (2.9)
9 United Republic of Tanzania	0.2 (2.6)	9 United Republic of Tanzania	0.3 (2.8)
10 Japan	0.2 (2.6)	10 Uganda	0.3 (2.8)
14 Uganda	0.1 (0.2)	26 Japan	0.1 (0.7)

b

Country	Exposed days/person in 2000-09	Country	Exposed days/person in 2010-19
1 Zambia	22.1	1 Zambia	28.8
2 Malawi	14.1	2 Democratic Republic of the Congo	24.3
3 Angola	11.8	3 Malawi	21.7
4 Democratic Republic of the Congo	11.8	4 Guatemala	17.7
5 Guatemala	10.7	5 Burundi	16.8
6 Burundi	10.2	6 Zimbabwe	16.2
7 Zimbabwe	7.9	7 Angola	15.9
8 Mexico	7.8	8 Rwanda	14.9
9 Honduras	7.0	9 Cambodia	12.1
10 Mozambique	7.0	10 Mozambique	10.9
18 Rwanda	4.8	13 Mexico	9.5
40 Cambodia	1.6	15 Honduras	9.0

c

Country	Exposed persons in 2000-09 (%)	Country	Exposed persons in 2010-19 (%)
1 Indonesia	72.1 (9.5)	1 Indonesia	101.0 (9.4)
2 Mexico	68.1 (8.9)	2 Brazil	89.9 (8.4)
3 Brazil	64.7 (8.5)	3 Mexico	73.2 (6.8)
4 Russian Federation	56.6 (7.4)	4 Democratic Republic of the Congo	72.6 (6.8)
5 Democratic Republic of the Congo	43.5 (5.7)	5 Russian Federation	64.6 (6.0)
6 Japan	34.6 (4.5)	6 China	55.3 (5.2)
7 China	32.6 (4.3)	7 United States of America	36.0 (3.4)
8 Myanmar	22.6 (3.0)	8 Uganda	32.1 (3.0)
9 Thailand	20.8 (2.7)	9 Thailand	28.3 (2.6)
10 United States of America	17.4 (2.3)	10 United Republic of Tanzania	25.3 (2.4)
12 Uganda	16.0 (2.1)	12 Japan	22.3 (2.1)
13 United Republic of Tanzania	15.3 (2.0)	17 Myanmar	16.9 (1.6)

Fig. S13.

Leading countries with greatest annual population co-exposure to heatwave and substantial fire-sourced air pollution (SFAP) from 2000 to 2019, using three different exposure metrics. (a) annual person days (billion); (b) annual days per person; (c) annual persons (million).

Table S1.

Trends in proportions of person-days exposed to substantial fire-sourced air pollution (SFAP) within heatwave-exposed days and heatwave within SFAP-exposed days from 2000 to 2019.

Continent	Proportions of person-days exposed to SFAP within heatwave-exposed days				Proportions of person-days exposed to heatwave within extreme SFAP-exposed days			
	2000-09	2010-19	Change per decade	P for trend	2000-09	2010-19	Change per decade	P for trend
Global	3.4	4.3	0.7	0.01	10	17.2	6.6	<0.001
Africa	9.5	11.5	1.3	0.113	6.9	13.4	6	<0.001
Asia	1.6	1.9	0.3	0.444	9.8	16.4	6.1	0.001
Europe	1.1	1.3	-0.4	0.591	26.3	38.4	3.7	0.697
North America	8.1	8.1	0.4	0.850	35	49	11.6	0.018
Oceania	5.8	2	-3.1	0.168	17.7	14.3	-0.6	0.910
South America	10.8	10.9	0.3	0.857	9.1	18.3	9.1	<0.001

Table S2.

Annual average number of persons exposed to heatwave and substantial fire-sourced air pollution (SFAP), stratified by continent.

Continent	Annual persons exposed to heat (×10^6)				Annual persons exposed to SFAP (×10^6)			
	2000-09	2010-19	Change per decade	P for trend	2000-09	2010-19	Change per decade	P for trend
Global	6415.3	7316.7	910.4	<0.001	2006.5	2071.8	124.9	0.067
Africa	892.9	1170.7	279.7	<0.001	512.6	596.7	83.5	<0.001
Asia	3950.8	4441.1	494.8	<0.001	873.6	802.1	-20.4	0.764
Europe	689.2	703.3	13.9	<0.001	127.7	96.2	-16.3	0.555
North America	504.0	562.9	58.4	<0.001	167.8	217.7	46.1	0.041
Oceania	28.8	33.4	4.6	<0.001	16.6	16.6	1.7	0.495
South America	349.5	405.2	59.0	<0.001	308.2	342.4	30.4	0.096

Table S3.

Annual average number of person-days exposed to heatwave and substantial fire-sourced air pollution (SFAP), stratified by continent.

Continent	Annual person-days exposed to heat ($\times 10^8$)				Annual person-days exposed to SFAP ($\times 10^8$)			
	2000-09	2010-19	Change per decade	P for trend	2000-09	2010-19	Change per decade	P for trend
Global	1815.1	2834.4	994.6	<0.001	624.5	714.6	80.4	0.017
Africa	231.2	454.2	223.6	<0.001	311.2	381.0	61.0	<0.001
Asia	1145.4	1727.1	567.9	<0.001	177.5	194.7	16.1	0.59
Europe	210.5	260.0	49.4	0.006	9.8	6.2	-4.3	0.157
North America	138.0	219.4	73.6	<0.001	29.9	36.5	8.5	0.212
Oceania	7.5	12.0	5.1	<0.001	1.8	1.6	0.1	0.858
South America	82.4	161.6	75.1	<0.001	94.2	94.6	-0.9	0.933

Table S4.

Annual average number of days exposed to heatwave and substantial fire-sourced air pollution (SFAP) per person, stratified by continent.

Continent	Annual days per person exposed to heat				Annual days per person exposed to SFAP			
	2000-09	2010-19	Change per decade	P for trend	2000-09	2010-19	Change per decade	P for trend
Global	27.9	38.6	10.3	<0.001	9.6	9.8	0.0	0.972
Africa	25.2	38.4	13.2	<0.001	34.3	32.6	-2.5	0.033
Asia	28.6	38.7	9.8	<0.001	4.4	4.4	-0.1	0.903
Europe	30.5	37.0	6.4	0.012	1.4	0.9	-0.6	0.147
North America	27.1	38.9	10.4	<0.001	5.9	6.5	0.9	0.471
Oceania	25.5	35.3	11.3	<0.001	6.4	4.6	-0.8	0.726
South America	22.2	39.3	16.0	<0.001	25.5	23.1	-2.7	0.357

Table S5.

Decomposition of decadal changes in co-exposure person-days into population growth and exposure intensity components.

Region	Change per decade ($\times 10^8$)	Contribution of change per decade		Percentage contribution*	
		Population component	Exposure component	Population (%)	Exposure (%)
**Continent**					
Africa	27.1	10.5	16.6	38.7	61.3
Asia	13.4	2.3	11.1	17.3	82.7
Europe	-0.4	-0.1	-0.3	22.5	77.5
North America	6.7	2.0	4.7	29.7	70.3
Oceania	8.3	1.5	6.8	18.2	81.8
South America	0.0	0.0	-0.1	-172.7	272.7
**HDI group**					
Low HDI	21.7	8.8	12.9	40.6	59.4
Medium HDI	9.2	2.6	6.6	28.6	71.4
High HDI	20.6	4.3	16.3	20.8	79.2
Very high HDI	3.6	0.5	3.0	14.7	85.3
**Income group**					
Low income	19.9	8.2	11.8	40.9	59.1
Lower middle income	11.6	3.4	8.2	29.2	70.9
Upper middle income	22.6	4.5	18.2	19.7	80.3
High income	1.0	0.3	0.7	28.2	71.8
**Global**	55.1	16.3	38.8	29.5	70.5

*Negative percentages indicate components offsetting the overall change. Values may exceed 100% when opposing components partially cancel each other and the total change is small.

Table S6.

Global population co-exposure to heatwave and substantial fire-sourced air pollution (SFAP) from 2000 to 2019, using an alternative heatwave definition (90th percentile + ≥15 °C threshold).

	Annual person days co-exposure to heat and SFAP ($\times 10^8$)					Annual days co-exposure to heat and SFAP per person					Annual persons co-exposure to heat and SFAP ($\times 10^6$)				
	2000-09	2010-19	2000-19	Change per decade	P for trend	2000-09	2010-19	2000-19	Change per decade	P for trend	2000-09	2010-19	2000-19	Change per decade	P for trend
**Continent**															
Africa	21.9	51.4	36.6	27.1	<0.001	2.4	4.4	3.4	1.7	<0.001	199.7	315.6	257.7	109.2	<0.001
Asia	17.9	31.8	24.9	13.4	0.0	0.5	0.7	0.6	0.3	0.0	269.7	376.9	323.3	94.8	0.0
Europe	2.0	3.5	2.8	-0.4	0.8	0.3	0.5	0.4	-0.1	0.8	53.0	53.8	53.4	-7.2	0.7
North America	11.5	18.2	14.9	6.7	0.1	2.3	3.2	2.8	1.0	0.2	116.8	155.2	136.0	38.3	0.0
Oceania	0.4	0.3	0.3	0.0	0.9	1.4	0.8	1.1	-0.3	0.6	7.3	6.1	6.7	0.7	0.8
South America	8.6	16.9	12.8	8.0	0.0	2.5	4.4	3.4	1.8	0.0	104.7	149.6	127.2	45.4	0.0
**HDI group**															
Low HDI	15.3	38.8	27.1	21.7	<0.001	2.5	4.7	3.6	2.0	<0.001	141.1	236.0	188.5	89.1	<0.001
Medium HDI	10.6	20.7	15.6	9.2	<0.001	0.6	1.0	0.8	0.4	0.0	117.2	160.8	139.0	44.8	0.0
High HDI	27.3	47.1	37.2	20.3	0.0	1.1	1.7	1.4	0.6	0.0	322.4	457.9	390.2	134.4	0.0
Very high HDI	9.2	15.4	12.3	3.4	0.1	0.7	1.0	0.8	0.2	0.2	169.9	201.3	185.6	12.9	0.6
**Income group**															
Low income	13.6	35.3	24.5	19.9	<0.001	3.0	5.9	4.4	2.6	<0.001	122.9	207.4	165.1	79.4	<0.001
Lower middle income	12.6	25.4	19.0	11.4	<0.001	0.5	0.9	0.7	0.3	0.0	163.1	235.7	199.4	70.5	0.0
Upper middle income	32.3	56.7	44.5	22.4	0.0	1.3	2.1	1.7	0.7	0.0	383.6	519.4	451.5	128.7	0.0
High income	3.8	4.7	4.3	0.9	0.5	0.3	0.4	0.4	0.0	0.7	81.6	94.3	87.9	2.3	0.9
**Global**	62.4	122.1	92.2	54.7	<0.001	1.0	1.7	1.3	0.6	<0.001	751.3	1057.1	904.2	281.1	<0.001

Table S7.

Global population co-exposure to heatwave and substantial fire-sourced air pollution (SFAP) from 2000 to 2019, with population fixed at 2010 levels.

	Annual person days co-exposure to heat and SFAP ($\times 10^8$)					Annual days co-exposure to heat and SFAP per person					Annual persons co-exposure to heat and SFAP ($\times 10^6$)				
	2000-09	2010-19	2000-19	Change per decade	P for trend	2000-09	2010-19	2000-19	Change per decade	P for trend	2000-09	2010-19	2000-19	Change per decade	P for trend
**Continent**															
Africa	25.9	44.7	35.3	16.3	<0.001	2.5	4.3	3.4	1.6	<0.001	233.0	278.2	255.6	38.8	0.009
Asia	19.0	30.3	24.6	10.8	0.037	0.4	0.7	0.6	0.3	0.037	283.4	361.6	322.5	66.4	0.076
Europe	2.0	3.5	2.8	-0.3	0.870	0.3	0.5	0.4	0.0	0.870	52.4	54.4	53.4	-6.1	0.725
North America	12.6	17.3	14.9	4.7	0.205	2.3	3.2	2.8	0.9	0.205	126.9	148.2	137.5	21.6	0.230
Oceania	0.4	0.2	0.3	-0.1	0.595	1.4	0.8	1.1	-0.3	0.595	8.1	5.5	6.8	-0.7	0.788
South America	9.7	16.6	13.1	6.6	0.015	2.5	4.2	3.3	1.7	0.015	118.8	152.6	135.7	35.4	0.051
**HDI group**															
Low HDI	18.6	33.0	25.8	12.6	0.001	2.6	4.6	3.6	1.8	0.001	167.1	205.1	186.1	32.4	0.002
Medium HDI	11.7	19.3	15.5	6.7	0.004	0.6	1.0	0.8	0.3	0.004	128.2	149.8	139.0	23.2	0.066
High HDI	29.6	44.9	37.3	15.9	0.013	1.1	1.6	1.4	0.6	0.013	350.4	444.9	397.6	95.2	0.017
Very high HDI	9.7	15.3	12.5	2.9	0.148	0.7	1.0	0.8	0.2	0.147	176.2	199.6	187.9	4.8	0.832
**Income group**															
Low income	16.6	29.9	23.3	11.5	0.001	3.1	5.6	4.4	2.2	0.001	146.4	179.7	163.1	28.3	0.001
Lower middle income	14.0	23.6	18.8	8.2	0.001	0.6	0.9	0.7	0.3	0.001	179.6	222.3	200.9	40.4	0.044
Upper middle income	34.9	54.5	44.7	17.6	0.007	1.3	2.0	1.7	0.7	0.008	411.6	505.4	458.5	88.9	0.016
High income	4.0	4.6	4.3	0.6	0.613	0.3	0.4	0.4	0.1	0.613	84.8	92.7	88.8	-2.3	0.907
**Global**	69.6	112.6	91.1	38.0	<0.001	1.0	1.6	1.3	0.5	<0.001	822.5	1000.5	911.5	155.4	0.005
